# Fecal metabolite profiling identifies critically ill patients with increased 30-day mortality

**DOI:** 10.1126/sciadv.adt1466

**Published:** 2025-06-04

**Authors:** Alexander P. de Porto, Nicholas P. Dylla, Matthew Stutz, Huaiying Lin, Maryam Khalid, Michael W. Mullowney, Jessica Little, Amber Rose, David Moran, Mary McMillin, Victoria Burgo, Rita Smith, Che Woodson, Carolyn Metcalfe, Ramanujam Ramaswamy, Christopher Lehmann, Matthew Odenwald, Nadeem Bandealy, Jack Zhao, Marie Kim, Emerald Adler, Anitha Sundararajan, Ashley Sidebottom, John P. Kress, Krysta S. Wolfe, Eric G. Pamer, Bhakti K. Patel

**Affiliations:** ^1^ Duchossois Family Institute, University of Chicago, Chicago, IL 60637, USA.; ^2^ Department of Internal Medicine, Amsterdam UMC, University of Amsterdam, Amsterdam, Noord Holland 1081 HV, Netherlands.; ^3^ Ecolab, Saint Paul, MN 55102, USA.; ^4^ Division of Pulmonary and Critical Care Medicine, John H. Stroger Jr. Hospital of Cook County, Chicago, IL 60612, USA.; ^5^ New York Institute of Technology College of Osteopathic Medicine, Glen Head, NY 11545, USA.; ^6^ Department of Biological Sciences, Northern Illinois University, DeKalb, IL 60115, USA.; ^7^ Borch Department of Medicinal Chemistry and Molecular Pharmacology, Purdue University, West Lafayette, IN 47907, USA.; ^8^ Department of Medicine, Section of Infectious Diseases and Global Health, University of Chicago Medicine, Chicago, IL 60637, USA.; ^9^ Department of Medicine, Section of Gastroenterology, Hepatology and Nutrition, University of Chicago Medicine, Chicago, IL 60637, USA.; ^10^ Department of Medicine, Section of Pulmonary and Critical Care Medicine, University of Chicago Medicine, Chicago, IL 60637, USA.; ^11^ Division of Epidemiology and Biostats, School of Public Health, University of Illinois Chicago, Chicago, IL 60607, USA.; ^12^ Tempus AI, Chicago, IL 60654, USA.

## Abstract

Critically ill patients admitted to the medical intensive care unit (MICU) have reduced intestinal microbiota diversity and altered microbiome-associated metabolite concentrations. Metabolites produced by the gut microbiota have been associated with survival of patients receiving complex medical treatments and thus might represent a treatable trait to improve clinical outcomes. We prospectively collected fecal specimens, defined microbiome compositions by shotgun metagenomic sequencing, and quantified microbiota-derived fecal metabolites by mass spectrometry from 196 critically ill patients admitted to the MICU for non–COVID-19 respiratory failure or shock to correlate microbiota features and metabolites with 30-day mortality. Microbiota compositions of the first fecal sample after MICU admission did not independently associate with 30-day mortality. We developed a metabolic dysbiosis score (MDS) that uses fecal concentrations of 13 microbiota-derived metabolites, which predicted 30-day mortality independent of known confounders. The MDS complements existing tools to identify patients at high risk of mortality by incorporating potentially modifiable, microbiome-related, independent contributors to host resilience.

## Teaser

Fecal metabolic dysbiosis is independently associated with 30-day mortality in critically ill patients.

## INTRODUCTION

Critically ill patients are often diagnosed with syndromes such as acute respiratory distress syndrome (ARDS) or sepsis. There is heterogeneity within these syndromes with respect to their pathogenesis. This biological heterogeneity has resulted in variable responses to treatments in clinical trials (*1*, *2*). Precision medicine targets interventions to biological states rather than syndromes and has the potential to improve therapeutic responses. The aim of precision medicine is to facilitate causal interference with a treatable trait, defined by Maslove *et al.* (*3*) as a specific physiological derangement characterized by biomarkers that portend a predictable response to a particular therapy.

Several studies have looked at associations between fecal microbiome taxonomic profiles and mortality in critically ill patients to identify a treatable trait. Low microbiome diversity and richness are common in intensive care unit (ICU) patients (*4*, *5*), and a low diversity, or a reduction in diversity during ICU stay, has been associated with increased mortality (*6*, *7*), albeit inconsistently (*8*, *9*). The microbiomes of critically ill patients have reduced abundances of commensal bacterial species and expanded populations of pathobionts during the ICU stay (*9*–*13*). *Enterococcus* domination has been associated with an increased risk of death or infection in ICU patients (*4*, *8*, *9*). Moreover, Enterobacterales enrichment is associated with the development of nosocomial infections due to a disturbance in myeloid cell responses and amplified systemic inflammation (*5*, *8*), but a direct link with mortality was not demonstrated.

Disturbances in the fecal metabolite concentrations may represent another microbiome-related treatable trait. However, data on the relationship between fecal metabolite concentrations and clinical outcome parameters are limited. Short-chain fatty acids (SCFAs), bile acids, and tryptophan metabolites are among the major groups of microbiota-derived metabolites (*14*). Admission to the ICU is associated with disturbances in fecal microbiome-derived metabolites, especially after treatment with antibiotics (*15*). A retrospective study linked low fecal levels of propionate and acetate (both SCFAs) to mortality (*16*). Also, low fecal indole-3-propionic acid (a tryptophan metabolite) is associated with higher disease severity and mortality in critically ill patients with sepsis (*17*). Moreover, in critically ill patients with COVID-19, the low concentration of three secondary bile acids (lithocholic acid, deoxycholic acid, and isodeoxycholic acid) and desaminotyrosine is associated with increased mortality (*18*).

To identify gut microbiome taxonomic and metabolic profiles associated with 30-day mortality after admission to the medical ICU (MICU), we performed a prospective observational cohort study. The microbial compositions and individual fecal metabolite concentrations in the first fecal sample after ICU admission did not correlate with 30-day mortality. However, we developed the metabolic dysbiosis score (MDS) using fecal concentrations of 13 metabolites in the first fecal sample after ICU admission. The MDS is independently associated with 30-day mortality in critically ill patients. Therefore, fecal metabolic dysbiosis may be a potentially treatable trait. Fecal metabolic profiles can provide guidance for microbiome-augmentation clinical trials by providing insights into the microbiome’s function and identifying patients with increased risk of mortality.

## RESULTS

### Basic characteristics

To study the association of the microbiome and microbiome-derived metabolites with overall 30-day mortality after admission to the MICU, we performed a prospective observational cohort study at The University of Chicago Medical Center. We enrolled 500 patients ≥18 years in need of respiratory support or a vasopressor upon admission to the MICU (fig. S1). Pregnant patients and patients with a prior cardiac arrest during admission of interest were excluded. We recently published on the microbiota of patients with COVID-19 and therefore excluded them from this analysis (*18*). One hundred ninety-six MICU patients without COVID-19 produced a fecal sample that was analyzable for metagenomics and metabolomics (fig. S1). For model validation, we split our data, stratifying for survival outcomes, into a training cohort (75%, 147 patients) and a validation cohort (25%, 49 patients) before any analyses to avoid data leakage (through data normalization, standardization, etc.). All data are from the training cohort unless mentioned otherwise.

The basic characteristics of our cohort are described in Table 1 and table S1. Overall, 30-day mortality was 30.6%. There was no significant difference between survivors and nonsurvivors in terms of age, sex, race, and overall comorbidity burden as measured by the Charlson comorbidity index (CCI). Although the overall CCI was not different, there was a higher percentage of patients with metastatic solid tumors in the nonsurvivors (*P* < 0.001) (table S1). Nonsurvivors more frequently had sepsis (*P* = 0.034) and ARDS (*P* = 0.001). Moreover, nonsurvivors had higher disease severity scores measured with the sequential organ failure assessment (SOFA) (*P* = 0.001) and acute physiology and chronic health evaluation (APACHE) II (*P* = 0.008) scores. For all analyses, we used the first fecal sample after study inclusion. Survivors and nonsurvivors had a similar time from MICU admission to first sample collection (*P* = 0.237) (fig. S2). Nonsurvivors more frequently received metronidazole (*P* = 0.017) in the 24 hours before fecal sample collection (Table 1).

**Table 1.** Description of training cohort baseline demographics, past medical history, severity of illness and antibiotic use. In the hospital antibiotic use up to 24 hours before fecal sample collection is reported. Categorical variables were compared using a two-tailed, chi-square test, while continuous variables were compared using the unpaired, two-tailed, two sample *t* test. Unadjusted *P* values are presented as exact values. APACHE, acute physiology and chronic health evaluation; IQR, interquartile range; SOFA, sequential organ failure assessment.

	Survivors	Nonsurvivors	*P*
Number of patients	102	45	
**Baseline characteristics**			
Age [median (IQR)]	63.50 (50.25, 70.00)	64.00 (54.00, 69.00)	0.756
Male (%)	54 (52.9)	27 (60.0)	0.54
Race (%)			0.085
African American	72 (70.6)	24 (53.3)	
Asian	1 (1.0)	0 (0.0)	
More than one race	2 (2.0)	3 (6.7)	
White, Hispanic	3 (2.9)	0 (0.0)	
White, non-Hispanic	24 (23.5)	18 (40.0)	
**Clinical characteristics**			
CCI [median (IQR)]	4.00 (3.00, 6.75)	5.00 (4.00, 7.00)	0.183
Body mass index [median (IQR)]	26.26 (21.72, 32.29)	24.10 (21.09, 28.06)	0.325
Acute respiratory distress syndrome (%)	17 (16.7)	20 (44.4)	0.001
Sepsis (%)	67 (65.7)	38 (84.4)	0.034
SOFA score [median (IQR)]	7.00 (4.00, 11.00)	10.00 (6.00, 14.00)	0.001
APACHE II score [median (IQR)]	23.50 (19.00, 29.00)	27.00 (23.00, 32.00)	0.008
**Antibiotics**			
Penicillins (%)	16 (15.7)	12 (26.7)	0.182
Cephalosporins (%)	68 (66.7)	35 (77.8)	0.246
Carbapenems (%)	5 (4.9)	3 (6.7)	0.968
Vancomycin (%)	56 (54.9)	32 (71.1)	0.096
Metronidazole (%)	38 (37.3)	27 (60.0)	0.017
Macrolides (%)	20 (19.6)	7 (15.6)	0.724
Quinolones (%)	6 (5.9)	2 (4.4)	1
Other antiobiotics (%)	8 (7.8)	2 (4.4)	0.69
Clindamycin (%)	2 (2.0)	1 (2.2)	1
Aminoglycosides (%)	11 (10.8)	5 (11.1)	1
Doxycycline (%)	5 (4.9)	0 (0.0)	0.309
Trimethoprim-sulfamethoxazole (%)	11 (10.8)	3 (6.7)	0.632
Rifaximin (%)	5 (4.9)	7 (15.6)	0.065

### No taxonomic associations with 30-day mortality after accounting for confounders

**Fig. 1.**
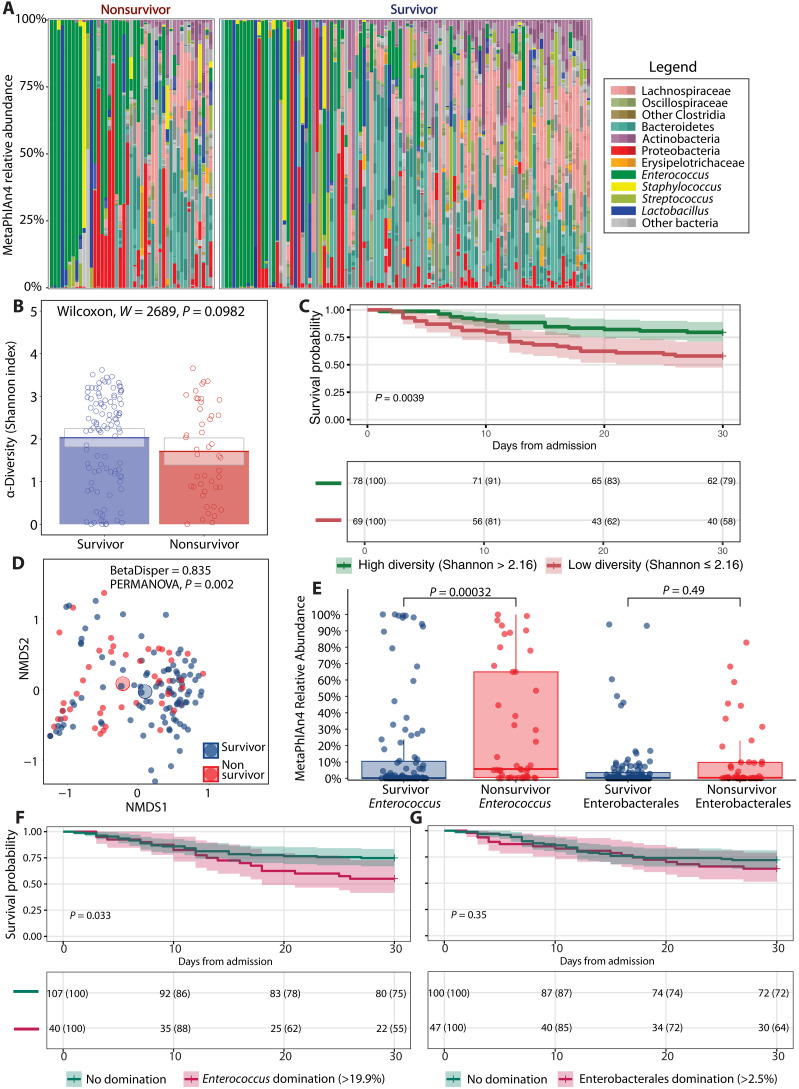
Shotgun metagenomic-derived diversity metrics and relevant taxa for survival outcomes. (**A**) Relative abundances for each patient where taxa are grouped and colored at varying, biologically relevant taxonomic levels. (**B**) Bar graph of alpha diversity, estimated by the Shannon index. (**C**) Kaplan-Meier curve with 30-day survival probabilities after admission to the ICU, stratifying patients according to their Shannon diversity values as determined by an optimal threshold analysis. (**D**) Beta diversity as determined by nonmetric multidimensional scaling (NMDS). (**E**) Box plot of relative abundances of pathogenic taxa. (**F**) Kaplan-Meier curve with 30-day survival probabilities after admission to the ICU, stratifying patients according to *Enterococcus* relative abundance status (genus level). (**G**) Thirty-day survival probabilities after admission to the ICU, stratifying patients according to Enterobacterales relative abundance status (order level). Bar graphs show average values for each group, while gray boxes denote 95% CIs. Box plots show interquartile ranges (IQRs) where the center bolded line represents the median and the whiskers (vertical lines) extend either to 1.5 times the IQR or to the minimum and maximum values, depending on whichever is closest to the median value. Kaplan-Meier survival analyses have time after ICU admission represented on the *x* axis in days with the probability of survival of each stratified group shown on the *y* axis. Survival probability is shown below each curve at 10-day intervals. Groups in (B) and (E) were compared using Wilcoxon rank sum, two-tailed unpaired tests where *P* values were adjusted (*q* values) for multiple comparisons via the Benjamini-Hochberg method. Groups in (C), (F), and (G) were compared using a log-rank test to assess significance. In (D), homogeneity of dispersion was assessed using the Bray-Curtis distance matrix and tested via a permutation analysis of variance (PERMANOVA) model.

We first analyzed taxonomic features of the first fecal sample after MICU admission to identify microbiome characteristics associated with 30-day mortality that could be treatable traits. The fecal taxonomic composition of both groups ranged from diverse to complete domination by pathobionts (Fig. 1A). The Shannon index, as a reflection of alpha diversity, was not different between survivors and nonsurvivors (Fig. 1B). However, patients with a high Shannon index ( >2.16) had a significantly (*P* = 0.004) higher survival probability compared to patients with a low Shannon index, as shown by a Kaplan-Meier survival curve (Fig. 1C). The threshold for the Shannon index was calculated using the Youden index, which optimizes both sensitivity and specificity for the binary outcome of 30-day mortality (fig. S3). The overall taxonomic composition (beta diversity), calculated through the Bray-Curtis dissimilarity metric, was different between survivors and nonsurvivors (*P* = 0.002) (Fig. 1D).

We also determined the relative abundance of *Enterococcus* (genus level) and Enterobacterales (order level) in survivors and nonsurvivors. *Enterococci* are Gram-positive bacteria that are associated with an increased risk of death or infection in ICU patients (*4*, *8*, *9*). Enterobacterales are Gram-negative bacteria for which expansion is associated with the development of nosocomial infections in ICU patients (*5*, *8*). The relative abundance of *Enterococcus* was significantly higher in nonsurvivors compared to survivors (*P* < 0.001) (Fig. 1E). There was no significant difference in relative abundance of Enterobacterales between groups (Fig. 1E). Patients dominated with *Enterococcus* (>19.9%) had a significantly (*P* = 0.033) lower survival probability compared to patients with no domination, as shown by a Kaplan-Meier survival curve (Fig. 1F). However, there was no difference in survival probability (*P* = 0.35) between patients dominated with Enterobacterales (>2.5%) compared with patients who were not dominated (Fig. 1G). Because there are no strict definitions of “pathogenic” relative abundances, we chose previously published abundance thresholds that exceed those seen in healthy donors for *Enterococcus* and Enterobacterales domination (*19*).

The difference in survival probability between patients with a low and high Shannon index and patients with a high and low relative abundance of *Enterococcus* suggests that these taxonomic features could be a treatable trait. However, there might be confounding factors. We therefore included the CCI as a measure of comorbidity burden and the SOFA score upon admission to the MICU as a measure of disease severity as confounders in addition to taxonomic features in a Cox proportional hazard model. We included the taxonomic features as a continuous and dichotomous variable. All variables within the Cox proportional hazard model fulfilled the proportional hazard assumption according to the Schoenfeld individual test (fig. S4). The Cox proportional hazard models show that every unit decrease in the Shannon index or a low diversity state (Shannon index ≤ 2.16) is not significantly associated with an increased risk of 30-day mortality (*P* = 0.719 and 0.158, respectively) (table S2). Diversity is therefore unlikely a relevant treatable trait to prevent 30-day mortality of critically ill patients. Likewise, every unit increase in *Enterococcus* relative abundance or *Enterococcus* domination (relative abundance > 19.9%) is not significantly associated with an increased risk of 30-day mortality (*P* = 0.161 and *P* = 0.121, respectively) (table S3).

To determine whether there are other specific taxonomic associations with 30-day mortality after MICU admission, we performed differential abundance analyses corrected and uncorrected for confounders (CCI and SOFA score) using MaAsLin2 (*20*). This multivariate analysis did not result in any significant associations at species, genus, and family levels. In conclusion, taxonomic features were not independently associated with 30-day mortality in patients admitted to the MICU.

### No association between single metabolites and 30-day mortality

**Fig. 2.**
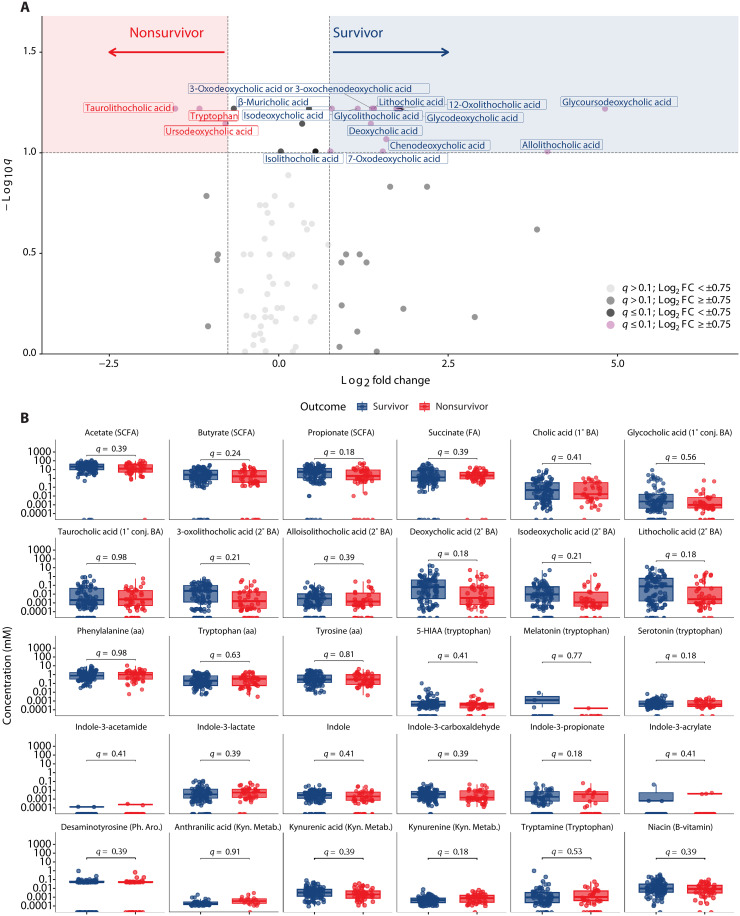
Qualitative and quantitative metabolomics across survivors and nonsurvivors. (**A**) Volcano analysis of qualitatively estimated metabolites, where values with *q* ≤ 0.1 are shown above the horizontal line and log_2_FC values greater than 0.75 or less than −0.75 fall in the red and blue shaded areas. (**B**) Metabolites were quantitatively calculated across survival groups. 5-HIAA, 5-hydroxyindoleacetic acid; aa, amino acid; BA, bile acid; Conj, conjugated; FA, fatty acid; Kyn. Metab, kynurenine metabolite; Ph.Aro, Phenolic Aromatic metabolite; SCFA, short chain fatty acid. In (A) and (B), Wilcoxon rank sum, two-tailed unpaired tests were performed, and *P* values were adjusted (*q* values) for multiple comparisons via the Benjamini-Hochberg method.

The microbiome produces a vast array of metabolites, which can affect human health (*14*). We therefore examined differences in fecal metabolite concentrations between survivors and nonsurvivors. We performed relative quantification of 83 fecal metabolites including SCFAs, primary and secondary bile acids, and tryptophan metabolites including indoles (Fig. 2A and table S4). No significant differences (Benjamini-Hochberg method adjusted *P* < 0.05) were found in fecal concentrations between survivors and nonsurvivors. However, 13 metabolites, all of which were bile acids, had a log_2_ fold change (log_2_FC) > 0.75 in survivors compared to nonsurvivors and an adjusted *P* < 0.1. Tryptophan, taurolithocholic acid, and ursodeoxycholic acid had a log_2_FC < −0.75 in survivors compared to nonsurvivors and an adjusted *P* < 0.1.

We also performed absolute quantification of 30 metabolites including SCFAs, primary and secondary bile acids, amino acids, and tryptophan metabolites (indoles, serotonin including derivatives, and kynurenine including derivatives). There were no significant differences (adjusted *P* < 0.05) in fecal concentrations of these metabolites between survivors and nonsurvivors on univariate analysis (Fig. 2B).

In conclusion, these results demonstrate no significant differences in single metabolite concentrations between survivors and nonsurvivors. However, there is a trend toward higher fecal concentrations of secondary bile acids in survivors.

### The MDS is predictive of and independently associated with 30-day mortality

We reasoned that a comprehensive model that integrates the fecal metabolite concentrations might be associated with 30-day mortality. We previously published that a fecal MDS (COVID-19 MDS), which consisted of three secondary bile acids (deoxycholic acid, lithocholic acid, and isodeoxycholic acid) and desaminotyrosine, was predictive of and independently associated with mortality of critically ill patients with COVID-19 (*18*). In our cohort of non–COVID-19 MICU patients, a high score (≥2 points) was significantly associated with higher mortality (*P* = 0.006) as compared to patients with a low score (<2 points), as shown by a Kaplan-Meier survival curve (fig. S5). Although the COVID-19 MDS has a high specificity for correctly classifying nonsurvivors in the current cohort, accuracy and sensitivity were low (Table 2). A Cox proportional hazard model that includes the COVID-19 MDS and confounders (CCI and SOFA), which fulfilled the proportional hazard assumption (fig. S4), was not associated with an increased risk of 30-day mortality (continuous *P* = 0.257 and dichotomous *P* = 0.07) (table S5). This suggests that the COVID-19 MDS may not be generalizable to non–COVID-19 MICU patients.

**Table 2.** Performance metrics of different models used to predict 30-day mortality after MICU admission in the training cohort. MDS, metabolic dysbosis score; ROC AUC, receiver operating characteristic area under the curve.

Model	ROC AUC	Accuracy (95% CI)	Sensitivity (95% CI)	Specificity (95% CI)
**Training cohort**				
COVID-19 MDS	0.617	0.537 (0.453–0.620)	0.412 (0.315–0.514)	0.822 (0.680–0.920)
Logistic regression	0.611	0.694 (0.619–0.768)	0.000 (0.0–0.0)	1.000 (1.0–1.0)
Random forest	0.991	0.966 (0.611–0.858)	0.911 (0.828–0.994)	0.990 (0.971–1.009)
Extreme gradient boosting	0.976	0.905 (0.937–0.995)	0.711 (0.579–0.843)	0.990 (0.971–1.009)
MDS model	0.856	0.837 (0.767–0.893)	0.892 (0.815–0.945)	0.711 (0.557–0.836)
**Validation cohort**				
COVID-19 MDS	0.663	0.531 (0.383–0.675)	0.412 (0.247–0.593)	0.8 (0.519–0.957)
Logistic regression	0.651	0.694 (0.565–0.825)	0	1
Random forest	0.720	0.735 (0.611–0.858)	0.467 (0.214–0.719)	0.853 (0.734–0.972)
Extreme gradient boosting	0.678	0.673 (0.542–0.805)	0.2 (−0.002–0.402)	0.882 (0.774–0.991)
MDS model	0.683	0.714 (0.567–0.834)	0.467 (0.212–0.734)	0.824 (0.655–0.932)

**Fig. 3.**
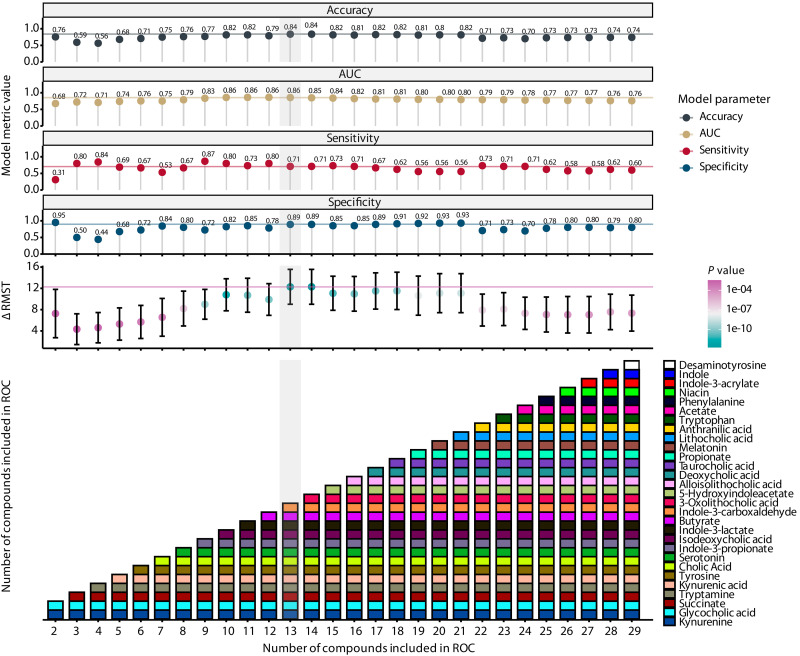
Model and survival metrics of the MDS. The top panels show accuracy, AUC, sensitivity, specificity, and the difference in RMST between low and high scores (as determined by an optimal threshold analysis) of each model. The lowest panel shows the number of compounds sequentially added into the model (*x* axis) with the compounds noted in each iteration (*y* axis and colored boxes). The order of compounds added into the scores was determined by increasing absolute β values from a ridge regression. RMST *P* values were determined using a log-rank test.

We sought to develop a model with better performance metrics. We built logistic regression, extreme gradient boosting, and random forest models using the absolute fecal metabolite concentrations to predict mortality (table S6). In addition, we built an MDS model based on optimal thresholds of fecal metabolite concentration (in millimolar) that were established by optimizing the Youden index in a receiver operating characteristic (ROC) analysis for each metabolite (table S7). Direction was established relative to the nonsurvivor group (table S7). To assess the variable importance of each metabolite, a ridge regression was performed to predict the survival outcome (table S7). Then, metabolites were included sequentially in an ROC analysis based on their variable importance, using beta coefficients as determined by the ridge regression, until all metabolites were used (Fig. 3). Indole-3-acetamide was not included in the model because only four values were above the detection limit (Fig. 2B). Similar to the COVID-19 MDS, for every patient, the score was determined by giving a point for every metabolite within the range of the concentration that was associated with nonsurvivors. The sum of all the points is the MDS. The threshold for the MDS was again calculated using the Youden index. For each combination of metabolites, model performance metrics—accuracy, specificity, sensitivity, and restricted mean survival time (RMST) difference—were assessed. The simplest but best-performing model was chosen and consisted of 13 metabolites (Fig. 3). The threshold was found to be at 7.5, meaning that a score > 7.5 represents significant metabolic dysbiosis. The relative fecal concentration of the 13 MDS metabolites in relation to the other 70 relatively quantified metabolites is shown in fig. S6.

The performance metrics of the four new models and the COVID-19 MDS in our training cohort and validation cohort are reported in Table 2. Basic characteristics of our validation cohort are shown in table S8. Within the validation cohort, the random forest and MDS models had comparable accuracy, sensitivity, and specificity and outperformed the COVID-19 MDS. The logistic regression and extreme gradient-boosting models lacked sensitivity compared to the random forest and MDS models. The 13 metabolites included in the MDS model largely overlap the metabolites that are most important for the random forest model, as determined by their Gini importance (fig. S7).

**Fig. 4.**
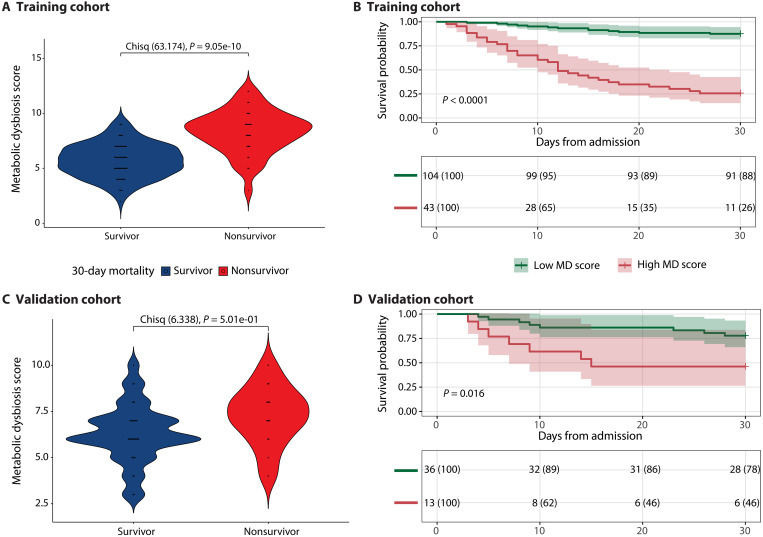
Evaluating the MDS for predicting survival outcomes on both the training and validation cohorts. Violin plots of the MDS for survivors and nonsurvivors in the training cohort (**A**) and validation cohort (**C**). Chisq, chi-square. Kaplan-Meier survival analyses starting from the day of admission to the ICU, stratifying patients according to their MDSs (as determined by an optimal threshold analysis) for the training cohort (**B**) and validation cohort (**D**). Chi-square tests were performed in (A) and (C) for integer values of the MDS. Kaplan-Meier survival analyses have time after ICU admission represented on the *x* axis in days with the probability of survival of each stratified group shown on the *y* axis. Survival probability is shown below each curve at 10-day intervals.

We chose to continue with the MDS because it is among the best-performing models and has greater interpretability, allowing for comparison between groups. Moreover, the MDS illuminates metabolite concentrations that are potentially relevant for 30-day mortality. Within the training cohort, the MDS is significantly higher in nonsurvivors compared to survivors (survivors: 5.74 with SD of 1.50 and nonsurvivors: 8.33 with SD of 1.91) (*P* < 0.001) (Fig. 4A). A Kaplan-Meier survival curve demonstrates a significant (*P* < 0.001) difference in survival probability between patients with a low MDS (score < 7.5) compared to patients with a high MDS (score ≥ 7.5) (Fig. 4B). After confirming the proportional hazard assumption (fig. S4), we found that for each unit increase in the MDS, the risk of 30-day mortality increases by a factor of 1.73 (*P* < 0.001) after controlling for confounders (Table 3). When including the MDS as a dichotomous variable in a Cox proportional hazard model, we found that metabolic dysbiosis based on an MDS ≥ 7.5 increases the risk of 30-day mortality by a factor of 8.66 (*P* < 0.001) (Table 3). Inverse causal effects might confound these results because the MDS was derived using all first fecal samples without excluding those that were produced relatively late after ICU admission (e.g., after 3 days of ICU admission). However, after restricting the analysis to fecal samples collected within 3 days after ICU admission (65% of total patients), the survival analysis and a Cox proportional hazard model in the training cohort showed similar results (fig. S8 and table S9). Within these patients, the MDS model had an accuracy of 0.84 [95% confidence interval (CI): 0.75 to 0.91], sensitivity of 0.88 (95% CI: 0.77 to 0.94), and a specificity of 0.75 (95% CI: 0.56 to 0.89).

**Table 3.** Cox proportional hazard ratio model including the MDS in the training cohort. Unadjusted *P* values were obtained from a likelihood ratio test and reported as exact values. SOFA, sequential organ failure assessment.

Characteristic	Hazard ratio	95% CI	*P* value
**Continuous**			
CCI	1.15	1.04–1.28	0.008
SOFA score	1.07	1.00–1.14	0.036
MDS	1.73	1.49–2.02	1.35 × 10^−12^
**Dichotomous**			
CCI	1.09	0.99–1.21	0.071
SOFA score	1.07	1.0–1.15	0.069
MDS			
Low MDS (<7.5)			
High MDS (>7.5)	8.66	4.42–17.0	3.12 × 10^−10^

Within the validation cohort, the MDS is not significantly different between survivors and nonsurvivors (*P* = 0.5) (Fig. 4C), although the mean is higher in nonsurvivors (survivors: 6.29 with SD of 1.57 and nonsurvivors: 7.27 with SD of 1.58). The Kaplan-Meier survival curve demonstrates a significant (*P* = 0.01) difference in survival probability between patients with a low MDS (score < 7.5) compared to patients with a high MDS (Fig. 4D). In a Cox proportional hazard model, we found that for each unit increase in the MDS, the risk of 30-day mortality increases by a factor of 1.21, although this is not significant (*P* = 0.289) (Table 4). When including the MDS as a dichotomous variable, having metabolic dysbiosis increases the risk of 30-day mortality by a factor of 1.88, although not significant (*P* = 0.291) (Table 4). The results of the validation cohort show the same trends as the training cohort but were unable to demonstrate significance due to the smaller sample size and support the need to validate the MDS in a larger, independent cohort.

**Table 4.** Cox proportional hazard ratio model including the MDS in the validation cohort. Unadjusted *P* values were obtained from a likelihood ratio test and reported as exact values. CCI, Charlson comorbidity index; MDS, metabolic dysbiosis score.

Characteristic	Hazard ratio	95% CI	*P* value
**Continuous**			
CCI	1.15	0.94–1.40	0.188
SOFA score	1.12	0.98–1.28	0.111
MDS	1.21	0.85–1.74	0.289
**Dichotomous**			
CCI	1.15	0.94–1.42	0.176
SOFA score	1.11	0.96–1.27	0.158
MDS			
Low MDS (<7.5)			
High MDS (>7.5)	1.88	0.58–6.05	0.291

In conclusion, the MDS is a score consisting of 13 fecal metabolites that can accurately predict 30-day mortality in our heterogeneous cohort of critically ill patients. The independent association with 30-day mortality suggests that the metabolites within the MDS are potential candidates (i.e., treatable traits) for intervention to improve outcomes in critically ill patients but further validation is needed.

### Taxonomic parameters are not an accurate reflection of fecal metabolic dysbiosis

**Fig. 5.**
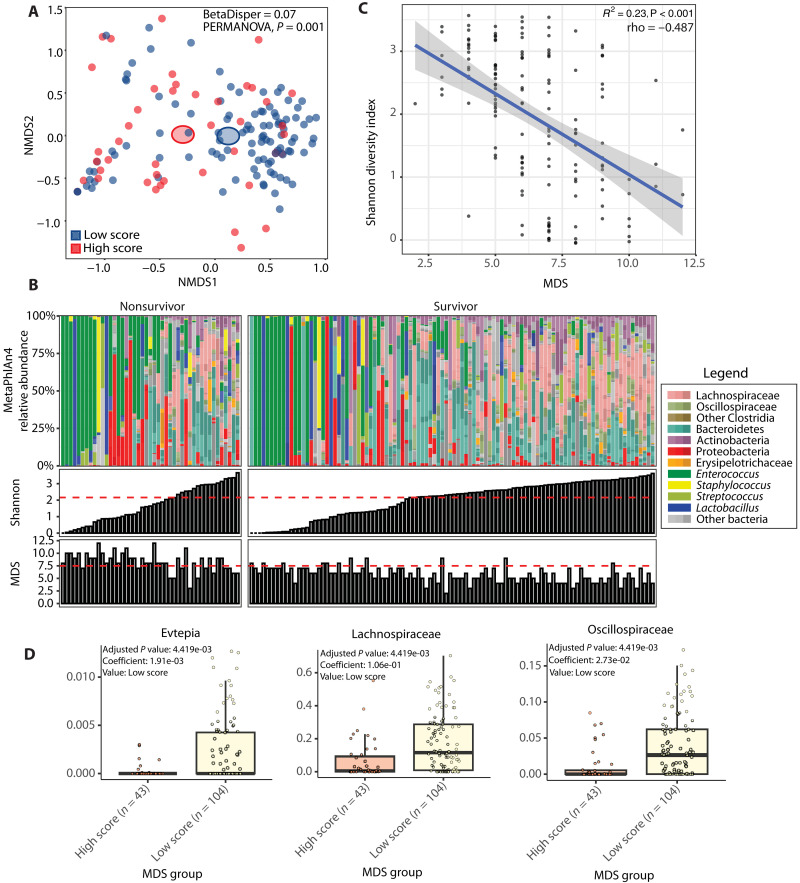
Fecal taxonomic and metabolic correlations. (**A**) Nonmetric multidimensional scaling plot based on Bray-Curtis distance metric. Each dot represents an individual patient color coded by its MDS. (**B**) In the top panel, relative abundances for each patient are shown where taxa are grouped and colored at varying, biologically relevant taxonomic levels. The panels below show the Shannon diversity index and MDS, respectively, for each individual patient. Horizontal lines represent the threshold determined by the Youden index that optimized sensitivity and specificity for the binary outcome of 30-day mortality. (**C**) Plot showing the correlation between the Shannon diversity index on the *y* axis and the MDS on the *x* axis. (**D**) Box plots with results from MaAsLin2 differential abundance analysis at the family level uncorrected for confounders. The *y* axis shows the relative abundance, and the *x* axis shows the MDS group. In (A), homogeneity of dispersion was assessed using the Bray-Curtis distance matrix and tested via a PERMANOVA. In (C), each dot represents an individual patient. The regression line is shown, and the gray bar around the regression line represents 95% CI. *R*^2^ is calculated from linear regression, while rho is Spearman statistics with its corresponding *P* values to show the strength of correlation. In (D), box plots show IQRs (75%: third quartile and 25%: first quartile) where the center bolded line represents the median. The upper whisker extends from the third quartile to the largest value no further than 1.5 × IQR from the third quartile. The lower whisker extends from the first quartile to the smallest value at most 1.5 × IQR of the first quartile.

Our results show that alpha-diversity is not related to 30-day mortality after MICU admission in the training cohort, whereas metabolic dysbiosis, defined as an MDS > 7.5, is. Although patients with a low and high MDS cluster together on a taxonomic multidimensional scaling plot (Fig. 5A), 24.5% of patients have a low Shannon diversity index but are not deemed in a state of metabolic dysbiosis, and 6.8% of patients have a high Shannon diversity index but deemed in a state of metabolic dysbiosis (Fig. 5B). Moreover, although there is a significant correlation between the Shannon index and the MDS, this correlation is moderate [coefficient of determination (*R*^2^) = 0.23, rho = −0.487, and *P* < 0.001] (Fig. 5C). Differential abundance analysis using MaAsLin2 without correction of covariates shows that members of the family of Evtepia, Lachnospiraceae, and Oscillospiraceae are significantly associated with a low MDS (Fig. 5D). However, when we correct for covariates, these associations no longer remain significant. A lack of correlation between fecal metabolite concentrations and microbiome’s abundance of metabolic genes has been previously demonstrated and suggests that exogenous factors, such as nutrient availability, affect the MDS (*21*). In conclusion, taxonomic parameters are not correlated with the degree of fecal metabolic dysbiosis.

## DISCUSSION

Fecal metabolic dysbiosis, determined by quantitation of 13 fecal metabolites, is independently associated with 30-day mortality after MICU admission in our cohort. Therefore, fecal metabolic dysbiosis represents a potentially treatable trait to improve survival in heterogeneous critically ill patients. The MDS may serve as a biomarker to identify patients who might benefit from correction of fecal metabolic dysbiosis.

The metabolites included in the MDS belong to the families of SCFAs, bile acids, and tryptophan metabolites. The metabolites within these groups influence physiological processes with plausible relevance for survival. SCFAs are produced by the microbiome in the large intestine via fiber-derived glycan fermentation. The SCFA butyrate is a key energy source for colonocytes, prevents colonocyte autophagy, and tightens the intestinal epithelial cell barrier through the induction of tight junctions (*22*–*25*). These butyrate-associated effects help prevent the systemic spread of bacteria and proinflammatory bacterial products. Butyrate increases colonization resistance by maintaining an anaerobic environment and regulating the production of antimicrobial peptides (*26*). SCFAs have anti-inflammatory effects such as the induction of T regulatory cell differentiation (*27*–*29*). In addition, butyrate was associated with increased survival of mice receiving a fecal microbiota transplantation after sepsis with a four-member pathogen community (*30*). In a clinical environment, the prognostic significance of fecal SCFA concentration is uncertain. One retrospective study associated low fecal concentrations of SCFAs with mortality in ICU patients (*16*). Another prospective study among 44 patients with sepsis did not find an association between fecal SCFA levels and mortality (*31*). Primary bile acids are mainly essential for the absorption of lipids and fat-soluble vitamins by the gut and can be converted into secondary bile acids by the microbiome in the large intestine. Secondary bile acids provide colonization resistance to *Clostridium difficile* and *Citrobacter rodentium* (*32*–*34*). Moreover, some modified secondary bile acids have anti-inflammatory effects such as promoting T regulatory cell differentiation, inhibiting T helper 17 cell differentiation in the gut (*35*–*37*), and limiting cytokine production in innate immune cells (*38*, *39*). However, their systemic effects are less elucidated. Tryptophan, an essential amino acid, can be metabolized into indoles, tryptamine, serotonin, and kynurenine via different pathways. Whereas indoles are solely produced by the microbiome, the remaining aforementioned metabolites can be formed by the host as well (*40*). Indoles and kynurenine pathway metabolites signal through the aryl hydrocarbon receptor (*41*), a transcription factor widely expressed including by immune cells, in a ligand-specific fashion (*42*). Indoles are able to regulate T cell differentiation and cytokine production, improve intestinal barrier integrity, and increase antimicrobial peptide production, leading to colonization resistance against pathogens such as *C. rodentium* (*14*). Low fecal indole-3-propionic acid, a tryptophan metabolite, is associated with worse clinical outcomes in critically ill patients (*17*). Serotonin acts both in the central nervous system as a neurotransmitter and in the peripheral as a hormone (*43*). Within the central nervous system, serotonin regulates the stress response, body temperature, and autonomic neural activity. Peripherally, it is important for the regulation of gut motility, vascular tone, heart rate, and respiratory drive. Moreover, it influences innate and adaptive immune responses upon release by platelets after activation (*43*). To our knowledge, no association between fecal serotonin levels and outcome of patients on the ICU has been reported.

As mentioned above, the metabolites within the MDS influence a wide array of distinct and overlapping processes. The need for a score that reflects this complexity is confirmed by our analyses. Univariate analysis of individual fecal metabolite concentrations did not result in any significant differences between survivors and nonsurvivors after ICU admission. However, the MDS, which is based on a multivariate analysis of fecal metabolite concentrations, revealed a relationship between fecal concentrations of 13 metabolites and 30-day mortality after MICU admission. It is notable that in this context, relatively higher concentrations of metabolites were not always associated with survivorship. For example, an increase in indole-3-lactate was associated with mortality, contrary to the other two indoles in the score for which low concentrations were associated with mortality (indoles: indole-3-propionate and indole-3-carboxaldehyde). Similar patterns were also found in the group of nonindole tryptophan metabolites.

It is unclear whether the metabolites in the MDS directly influence 30-day mortality or whether they are merely a reflection of fecal metabolic dysbiosis. Ridge regression will penalize metabolites that covary, which could have resulted in penalizing important metabolites while ascribing importance to a metabolite that is just covarying but has no direct biological effect on mortality. However, similar metabolites were important for 30-day mortality prediction in the random forest model, which performed similarly compared to the MDS. Future research should evaluate causal mechanisms that underlie how individual metabolites are important for survival.

Our previously published COVID-19 MDS (*18*), which associated fecal dysbiosis using four metabolites with mortality in critically ill patients with COVID-19, had high specificity for 30-day mortality in our heterogeneous cohort but lower sensitivity and accuracy. Moreover, it was not independently associated with 30-day mortality in a Cox proportional hazard model. The need to include 13 metabolites in the score to increase sensitivity and accuracy might be a result of the heterogeneous patient population within the MICU. Disease-specific fecal metabolic dysbiosis profiles might contain fewer metabolites, as is the case for the patients with COVID-19, and differ on the basis of disease characteristics and the functional roles of the fecal metabolites. Because of the inclusion of 13 metabolites within the MDS, many different combinations are possible to reach the threshold for fecal metabolic dysbiosis and are likely an assembly of all these potential disease-specific fecal MDSs.

Selective decontamination of the digestive tract (SDD) to prevent ICU-acquired infections has proven to reduce mortality in settings with relatively low prevalence of multidrug-resistant pathogens (*44*–*46*). SDD is aimed at eradicating Gram-negative bacteria, *Staphylococcus aureus*, and yeast while preserving the anaerobic flora. However, it was shown that SDD causes loss of commensals including butyrate producers (*47*, *48*). In the light of this study, it may be that the positive effect of SDD on mortality is reduced by its impact on microbiome-derived metabolites. Further investigation should focus on the potentially negative effect of SDD on the production of microbiome-derived metabolites.

While the findings are of potential utility to a diverse cohort of critically ill patients, this study is not without its limitations. Patients were recruited at a single tertiary academic center, where the patient population might not be generalizable to all critically ill patients. The patient population studied here is heterogeneous, and results might not be applicable to specific patient cohorts. We included patients in need of respiratory support or a vasopressor, and thus, the results might not be representative of the entire MICU population. Also, patients who did not produce a fecal sample or a sample that was not analyzable for metagenomics and metabolomics were excluded from the study. Gastrointestinal dysmotility is very frequent in the ICU (reported to be present in up to 70% of patients) and explains the high percentage of patients that did not produce a sample (*49*–*51*). The time between ICU admission and sample collection was not an exclusion factor to increase clinical utility and power of the study. This might result in inverse causal effects. However, 85% of patients had a sample collected within 5 days after ICU admission (fig. S2), and there was no significant difference between the days of fecal sample collection after ICU admission between survivors and nonsurvivors. Moreover, we included the MDS in a Cox proportional hazard model combined with potential confounders (comorbidity and disease severity) to mitigate this effect. Although the results in the training cohort are similar to those in the validation cohort, they fall short of significance in the validation cohort likely due to the smaller sample size and the heterogeneity of our cohort. Overfitting of our training cohort could be another limitation. Other large validation cohorts are needed to confirm our findings. Last, inherent to the nature of this study, we cannot show causality between the metabolites in the MDS and the outcome.

We did not find any taxonomic associations with 30-day mortality after MICU admission. However, in other studies, a low alpha-diversity, or a reduction in alpha-diversity during ICU stay, has been associated with mortality (*6*, *7*), although not consistently (*8*, *9*).

*Enterococcus* domination is previously associated with mortality or infection in ICU patients (*8*, *9*). Although it was recently described that Enterobacterales enrichment in ICU patients is associated with the development of nosocomial infections, we did not see increased relative abundances of Enterobacterales in nonsurvivors. Moreover, there was no difference in survival between patients who were and were not dominated by Enterobacterales.

The discrepancy between alpha-diversity and fecal metabolic dysbiosis in their independent association with 30-day mortality is remarkable. There is only a moderate correlation between the two. This may be caused by pathway-specific gene regulation of bacterial taxa, diet during ICU stay, and host factors (*21*). Assessing correlations between pathway abundance using microbiome gene expression data and fecal metabolomics might be an interesting next step to disentangle this complex interaction.

In the past, large untargeted clinical trials using probiotics in the ICU showed no benefit on mortality (*52*–*54*). The reason for this might be that these trials did not select eligible ICU patients for treatment based on a biological state (*3*). Therefore, future randomized clinical trials aiming to reconstitute fecal metabolic dysbiosis should target patients based on their biological state to prevent unnecessary treatment and increase the likelihood of success. Our MDS could serve as a biomarker but needs confirmation in other independent cohorts of critically ill patients. Treatment of fecal metabolic dysbiosis could be performed through dietary intervention (prebiotics), the administration of bacterial consortia (probiotics), or directly via the oral administration of metabolites (*55*). When designing trials with probiotics, the high frequency of antibiotic use in the ICU needs to be considered. Moreover, the administration of probiotics to ICU patients has been associated with an increase in adverse events (*56*). This emphasizes the need to treat only patients who have fecal metabolic dysbiosis to minimize risks for patients who do not need treatment based on their biological state. Thought also needs to go into consortia design, to optimally reconstitute all metabolic disruptions unless the important disease-specific disruptions are known, as, for example, in patients with COVID-19. A potential downside of orally administered metabolites might be that they do not reach the distal colon, where butyrate and secondary bile acids have their primary effect. Moreover, there are concentration-dependent negative effects of metabolites. For example, high butyrate and secondary bile acid concentrations have been associated with procarcinogenic properties (*57*). Elevated serum bile acid concentrations are correlated with 28-day mortality in ICU patients without cirrhosis or primary cholestatic disorders (*58*).

In conclusion, we show that fecal metabolic dysbiosis in critically ill patients upon MICU admission is independently associated with 30-day mortality in this cohort, and the MDS could potentially be used as a biomarker to identify patients with fecal metabolic dysbiosis. Moreover, we identify fecal metabolites that may be a treatable trait to improve the survival of critically ill patients.

## MATERIALS AND METHODS

### Study design, patient enrollment, and specimen collection

The study was performed at the University of Chicago Medical Center in the United States. It was conducted as previously described (*18*) and is a prospective observational cohort study. This study was approved by the University of Chicago Institutional Review Board (ID: IRB20-1102) and has been registered at clinicaltrials.gov as National Clinical Trial no. 04552834. Patients with respiratory failure in need of respiratory support (high-flow nasal canula, noninvasive positive pressure ventilation, or invasive mechanical ventilation) or shock (defined by the receipt of vasoactive medication) were eligible for inclusion. Exclusion criteria were age < 18 years, pregnancy, and prior cardiac arrest during admission of interest. Informed consent was obtained from the patient or surrogate decision makers before enrollment. Enrollment began in September 2020 and concluded in May 2021. Fecal samples were collected as soon as possible following ICU admission, immediately refrigerated, and aliquoted and frozen at −80°C within 24 hours.

### Cohort selection

Five hundred patients were enrolled. After exclusion of patients with COVID-19 and patients who did not produce a sample analyzable for metagenomics and metabolomics, 196 patients remained. The 196-patient population was randomly split into stratified training (70%, 147) and validation (30%, 49) groups to account for class imbalance in survivorship. The training set patients/samples were used for all descriptive figures and model development, while the validation cohort/data were withheld for the validation of the MDS in addition to descriptive statistics of the cohort.

### Clinical data

Clinical data collection was performed by chart review. Sepsis was defined according to the sepsis 3 criteria (*59*) as the suspicion or documentation of an infection and an acute increase of ≥2 SOFA points. ARDS was defined according to the Berlin definition (*60*). However, if there was no arterial blood gas available, then we used an SpO_2_/FiO_2_ ≤ 315 at SpO_2_ ≤ 97% instead of the PaO_2_/FiO_2_ ratio (*61*). The SOFA, APACHE II, and CCI scores were used according to their original definition. Antibiotic use was obtained through a data extraction procedure of the electronic medical record. In the hospital, antibiotic use up to 24 hours before fecal sample collection is reported. Fasting status was defined as being in the hospital in 2 days before sample collection and having a “nothing by mouth” order and not receiving tube feeding. Time to stool sample is the number of days after MICU admission that the stool sample was collected. All clinical data were stored in the REDCap secure online database (version LTS 13.1.27).

### Fecal DNA extraction

DNA was extracted using the QIAamp PowerFecal Pro DNA Kit (QIAGEN). Before extraction, samples were subjected to mechanical disruption using a bead-beating method. Briefly, samples were suspended in a bead tube (QIAGEN) along with lysis buffer and loaded on a bead mill homogenizer (Fisherbrand, Beadmill). Samples were then centrifuged, and the supernatant was resuspended in CD2, a reagent that effectively removes inhibitors by precipitating non-DNA organic and inorganic materials including polysaccharides, cell debris, and proteins. DNA was purified routinely using a silica spin column filter membrane. By adding solution CD3, a high-concentration salt solution, DNA was bound selectively to the membrane, which was then eluted using elution buffer. DNA was quantified using Qubit.

### Shotgun metagenomics

Libraries were prepared using 100 ng of genomic DNA using the QIAseq FX DNA Library Prep (QIAGEN), with the exception of samples that have low DNA yield. For samples with ultralow DNA concentration (10 ng or less), FX enhancer reagent was used. Briefly, DNA was fragmented enzymatically using a nuclease, and the desired insert size was achieved by adjusting fragmentation conditions. Fragmented DNA was end repaired using an end-repair enzyme, and “As” were added to the 3′ ends to stage inserts for ligation. During the ligation step, Illumina-compatible unique dual-index adapters were attached to the inserts, and the prepared library was polymerase chain reaction (PCR) amplified. Amplified libraries were purified, and quality control was performed using a TapeStation (Agilent). Libraries were normalized by quantitative PCR. Normalized libraries were sequenced on an Illumina NextSeq 500 or NextSeq 1000 to generate 2 × 150 base pair reads with the target production of 5 to 10 million reads. Adapters were trimmed off the raw reads, and their quality was assessed and controlled using Trimmomatic v.0.39 (*62*). Reads that mapped to the human genome were removed by kneaddata v0.7.10. Taxonomy was profiled using MetaPhlAn4 (*63*) using the resultant high-quality reads. α-Diversity of fecal samples was calculated using the Shannon index via the R package vegan and visualized using the R package ggpirate. beta-Diversity was determined using the R package vegan and the Bray-Curtis distance metric.

### Metabolomics

Methods and assay information can be accessed at MetaboLights (*64*). Pentafluorobenzyl bromide–derivatized metabolites including SCFAs (butyrate, acetate, and propionate) and succinate were analyzed by negative ion collision–induced gas chromatography–mass spectrometry [(–)GC-CI-MS; Agilent 8890]. Bile acids were analyzed by negative mode liquid chromatography–electrospray ionization–quadrupole time-of-flight–MS [(−)LC-ESI-QTOF-MS; Agilent 6546]. Tryptophan-related metabolites were analyzed by positive mode LC–triple quadrupole–MS [(+)LC-ESI-QQQ-MS; Agilent 6547]. All compounds have been validated through retention time and fragmentation comparison to standards and available databases.

### Statistical and bioinformatic analysis

All analyses were conducted using the R statistical language [v4.2.3 (2023-03-15)] using the RStudio (v2023.06.1 + 524) integrated development environment. Clinical comparisons were performed using nonparametric statistics when values were continuous. For categorical values, a chi-square goodness-of-fit test was used. All clinical tables were generated using the R package gtsummary (*65*). All other continuous variables regressed against categorical variables were compared using a two-tailed, Wilcoxon rank sum test, from the R package rstatix, unless otherwise noted. All *P* values were adjusted (*q* value) to account for multiple comparisons following the Benjamini-Hochberg method. The *q* values ≤ 0.05 were deemed statistically significant. In cases of missing metabolite values (<20% missing), the R package mice was used to impute values following the predictive mean-matching method (*66*). The compound desaminotyrosine was not included in the data imputation, as it was analyzed in completeness (for all patients) after the original compounds were analyzed. Metabolites with more than 20% missing values were not included here. To compare relatively quantified metabolites, log_2_FC was calculated per compound using the median value across all samples. These log_2_FC values were then arranged by statistical significance when comparing the diversity groups (Kruskal-Wallis test) and visualized via a volcano plot using the R package EnhancedVolcano.

To build the MDS, ROC curves were generated using the R package cutpointr (*67*) to determine the optimal concentration of each quantified metabolite that best separates the survivors from nonsurvivors. ROC curves were optimized for the Youden index and bootstrap-stratified 10 times (*68*). For each metabolite, concentrations associated with survivors were given an individual score of 0, while concentrations of metabolites that were associated with nonsurvivors were given a score of 1. A matrix of patients by compounds was built. To determine the importance of each metabolite, a ridge regression (α = 0) was performed on this matrix, which predicted survival outcome (survivor versus nonsurvivor) using the R package glmnet (*69*). The MDS was iteratively built by adding the next important compound to the total score until all 30 compounds were included in the score. Model metrics [accuracy, area under the curve (AUC), sensitivity, and specificity] were calculated for each iteration using the R package pROC (*70*). In addition to model performance assessments, the R package survRM2 was used to determine the RMST for each iterative score between the survivors and nonsurvivors. Regression between numeric variables was performed using stat_poly_eq() from ggpmisc R package with *R*^2^ and *P* values printed out on a scatterplot. Spearman rho statistics was calculated using cor.test with the method selected to use Spearman under the base R statistic package.

Differentially abundant species, genus, and family between survivors and nonsurvivors were analyzed using the MaAsLin model (R package MaAsLin2). The base model did not include confounding variables, while the mixed model included the CCI and SOFA scores. A similar analysis was performed to detect the differentially abundant species, genus, and family between MDS-high and MDS-low cohorts.

The training dataset was trained with three additional models to compare to the MDS model. Logistic regression (sklearn, scikit-learn, v1.5.0), random forest, and extreme gradient-boosting (xgboost, v2.1.3) models were trained using the training dataset with five splits with two repeats, and the best-tuned model was applied to the test dataset to evaluate the performance of the model. Parameters tuned include but not limited to the following: For logistic regression—penalty: none, L1, L2, or elasticnet; C: 0.001, 0.01, 0.1, 1, 10, 100, and 1000; and L1_ratio: 0.25, 0.5, and 0.75. For random forest—n_estimators: 500, 1000, and 1500; max_depth: none, 15, 30, and 45; min_samples_split: 5, 10, and 15; min_samples_leaf: 2, 4, and 6; and max_features: sqrt, log_2_, and 0.3. Last, for extreme gradient boosting,—n_estimators: 500 and 1000; max_depth: 2, 4, and 6; learning_rate: 0.01 and 0.1; and min_child_weight: 1 and 3. Model performance was assessed using ROC AUC, accuracy, sensitivity, and specificity with 95% CI. Best-performing parameters are displayed in table S6.

All survival analyses and Cox proportional hazard models were built using the R package survival, for creating a survival object, and survminer for plotting survival curves. All variables included in the Cox proportional hazard models met the proportional hazard assumption according to the Schoenfeld residual test (fig. S4). Survival analysis started from the day of admission to the ICU.
