# Supplementary material for: Fecal metabolite profiling identifies critically ill patients with increased 30-day mortality
Source: Sci Adv. 2025 Jun 4;11(23):eadt1466. doi: 10.1126/sciadv.adt1466 (PMC12136028; doi:10.1126/sciadv.adt1466)
Supplement: Supplementary file 1 — Supplementary Materials and Methods Figs. S1 to S8 Tables S1 to S9 [file sciadv.adt1466_sm.pdf]

Science Advances
AAAS

Supplementary Materials for

Fecal metabolite profiling identifies critically ill patients with increased 30-day mortality

Alexander P. de Porto *et al.*

Corresponding author: Alexander P. de Porto, adeporto@bsd.uchicago.edu;
Eric G. Pamer, egpamer@bsd.uchicago.edu; Bhakti K. Patel, bpatel@bsd.uchicago.edu

Sci. Adv. **11**, eadt1466 (2025)
DOI: 10.1126/sciadv.adt1466

This PDF file includes:

Supplementary Materials and Methods
Figs. S1 to S8
Tables S1 to S9

Supplemental materials and methods for metabolomics analysis

As previously published the following methods for metabolomics analysis were used (18).

Metabolite Extraction from Fecal Material

Extraction solvent (80% methanol spiked with internal standards and stored at -80 °C) was added at a ratio of 100 mg of material/mL of extraction solvent in beadruptor tubes (Fisherbrand; 15-340-154). Samples were homogenized at 4 °C on a Bead Mill 24 Homogenizer (Fisher; 15-340-163), set at 1.6 m/s with 6 thirty-second cycles, 5 seconds off per cycle. Samples were then centrifuged at -10 °C, 20,000 x *g* for 15 min and the supernatant was used for subsequent metabolomic analysis.

(1) Metabolite Analysis using GC-nCI-MS and PFBBr Derivatization

For Short chain fatty acids the metabolite extract (100 µL) was added to 100 µL of 100 mM borate buffer (pH 10) (Thermo Fisher, 28341), 400 µL of 100 mM pentafluorobenzyl bromide (Millipore Sigma; 90257) in Acetonitrile (Fisher; A955-4), and 400 µL of *n*-hexane (Acros Organics; 160780010) in a capped mass spec autosampler vial (Microliter; 09-1200). Samples were heated in a thermomixer C (Eppendorf) to 65 °C for 1 hour while shaking at 1300 rpm. After cooling to RT, samples were centrifuged at 4°C, 2000 x *g* for 5 min, allowing phase separation. The hexanes phase (100 µL) (top layer) was transferred to an autosampler vial containing a glass insert and the vial was sealed. Another 100 µL of the hexanes phase was diluted with 900 µL of n-hexane in an autosampler vial. Concentrated and dilute samples were analyzed using a GC-MS (Agilent 7890A GC system, Agilent 5975C MS detector) operating in negative chemical ionization mode, using a HP-5MSUI column (30 m x 0.25 mm, 0.25 µm; Agilent Technologies 19091S-433UI), methane as the reagent gas (99.999% pure) and 1 µL split injection (1:10 split ratio). Oven ramp parameters: 1 min hold at 60 °C, 25 °C per min up to 300 °C with a 2.5 min hold at 300 °C. Inlet temperature was 280 °C and transfer line was 310 °C. A 10-point calibration curve was prepared with acetate (100 mM), propionate (25 mM), butyrate (12.5 mM), and succinate (50 mM), with 9 subsequent 2x serial dilutions. Data analysis was performed using MassHunter Quantitative Analysis software (version B.10, Agilent Technologies) and confirmed by comparison to authentic standards. Normalized peak areas were calculated by dividing raw peak areas of targeted analytes by averaged raw peak areas of internal standards.

(2) Bile Acid Analysis

Bile acids were analyzed using LCMS. The metabolite extract (75 µL) was added to prelabeled mass spectrometry autosampler vials (Microliter; 09-1200) and dried down completely under a nitrogen stream at 30 L/min (top) 1 L/min (bottom) at 30 °C (Biotage SPE Dry 96 Dual; 3579M). Samples were resuspended in 50:50 Water:Methanol (750 µL). Vials were added to a thermomixer C (Eppendorf) to resuspend analytes at 4 °C, 1000 rpm for 15 min with an infinite hold at 4 °C. Samples were then transferred to prelabeled microcentrifuge tubes and centrifuged at 4 °C, 20,000 x *g* for 15 min to remove insoluble debris. The supernatant (700 µL) was transferred to a fresh, prelabeled mass spectrometry autosampler vial. Samples were analyzed on a liquid chromatography system (Agilent 1290 infinity II) coupled to a quadrupole time-of-flight (QTOF) mass spectrometer (Agilent 6546), operating in negative mode, equipped with an Agilent Jet Stream Electrospray Ionization source. The sample (5 µL) was injected onto an XBridge© BEH C18 Column (3.5 µm, 2.1 x 100 mm; Waters Corporation, PN) fitted with an XBridge© BEH C18 guard (Waters Corporation, PN) at 45 °C. Elution started with 72% A (Water, 0.1% formic acid) and 28% B (Acetone, 0.1% formic acid) with a flow rate of 0.4 mL/min for 1 min and linearly

increased to 33% B over 5 min, then linearly increased to 65% B over 14 min. Then the flow rate was increased to 0.6 mL/min and B was increased to 98% over 0.5 min and these conditions were held constant for 3.5 min. Finally, re-equilibration at a flow rate of 0.4 mL/min of 28% B was performed for 3 min. The electrospray ionization conditions were set with the capillary voltage at 3.5 kV, nozzle voltage at 2 kV, and detection window set to 100-1700 *m/z* with continuous infusion of a reference mass (Agilent ESI TOF Biopolymer Analysis Reference Mix) for mass calibration. A ten-point calibration curve was used for quantitation. Data analysis was performed using MassHunter Profinder Analysis software (version B.10, Agilent Technologies) and confirmed by comparison with authentic standards. Normalized peak areas were calculated by dividing raw peak areas of targeted analytes by averaged raw peak areas of internal standards.

(3) Indole/Tryptophan Analysis

Indole-containing metabolites, B-vitamins and other targeted metabolites were analyzed by LC-MS/MS. The metabolite extract (400 µL) was added to pre-labeled microcentrifuge tubes. Samples were dried down completely using a Genevac EZ-2 Elite. Samples were resuspended in 100 µL of 50:50 Water:Methanol and added to an Eppendorf thermomixer® C at 4 ^{O}C, 1000 rpm for 15 min to resuspend analytes. Samples were then centrifuged at 4 ^{O}C, 20,000 x *g* for 15 min to remove insoluble debris. The supernatant (80 µL) was transferred to a fresh, prelabeled MS vial with inserts or 96 deep-well plate (Agilent 5065-4402). Samples were analyzed on an Agilent 1290 infinity II liquid chromatography system coupled to an Agilent 6470 triple quadrupole mass spectrometer, operating in positive mode, equipped with an Agilent Jet Stream Electrospray Ionization source. Each sample (2 µL) was injected into a Acquity UPLC HSS PFP column, 1.8 µm, 2.1 x 100 mm (Waters; 186005967) equipped with a Acquity UPLC HSS PFP VanGuard Pre- column, 100Å, 1.8 µm, 2.1 mm X 5 mm (Waters; 186005974) at 45 ^{O}C. Mobile phase A was 0.35% formic acid in Water and mobile phase B was 0.35% formic acid in 95:5 Acetonitrile:Water. The flow rate was set to 0.5 mL/min starting at 0% B held constant for 3 min, then linearly increased to 50% over 5 min, then linearly increased to 95% B over 1 min, and held at 100% B for the next 3 min. Mobile phase B was then brought back down to 0% over 0.5 min and held at 0% for re-equilibration for 2.5 min. The QQQ electrospray conditions were set with capillary voltage at 4 kV, nozzle voltage at 500 V, and Dynamic MRM was used with cycle time of 500 ms. Transitions were monitored in positive mode for 46 analytes (table on next slide). An 11-point calibration curve (ranging from 0.88 nM to 909 µM) was prepared for tryptophan, tyrosine, phenylalanine, serotonin, 5-HIAA, melatonin, tryptamine, kynurenine, kynurenic acid, anthranilic acid, and niacin. Data analysis was performed using MassHunter Quant software (version B.10, Agilent Technologies) and confirmed by comparison with authentic standards. Normalized peak areas were calculated by dividing raw peak areas of targeted analytes by averaged raw peak areas of internal standards.

Quality Control Samples

Control biological samples are used to evaluate all processes. Specifically, metabolite extraction efficiency and instrument performance. The samples are extracted with solvent containing known internal standard (IS) concentrations. These samples are processed and analyzed alongside project runs. Recovery, retention time and %CV are calculated for ISs. Method blanks are run, and these samples do not contain any metabolites and are included in datasets to indicate instrument noise for the *m/z* at the retention time extracted. A small aliquot of submitter samples are combined to

create a pooled quality control (PooledQC) sample that is run to determine if there are matrix impacts on our processes.

Quality Control Ions

All samples are extracted with solvent that includes deuterated or heavy atom standards with known concentrations to evaluate metabolite extraction efficiency and instrument performance. Quality control ions are tracked in all control samples and the submitted samples and analyzed for batch impact and intra-project variability.

Supplemental figures

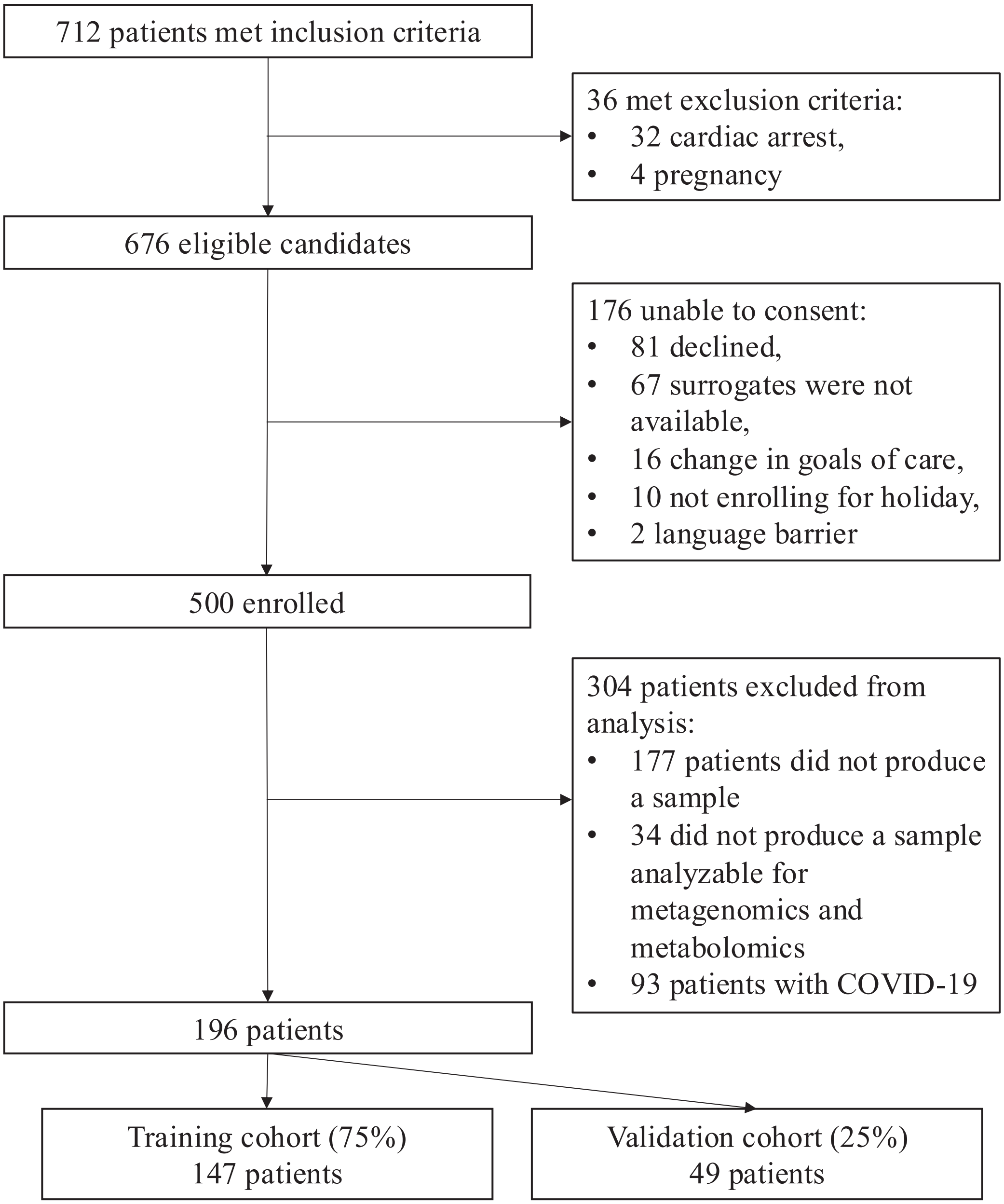

Fig. S1. Consort diagram.

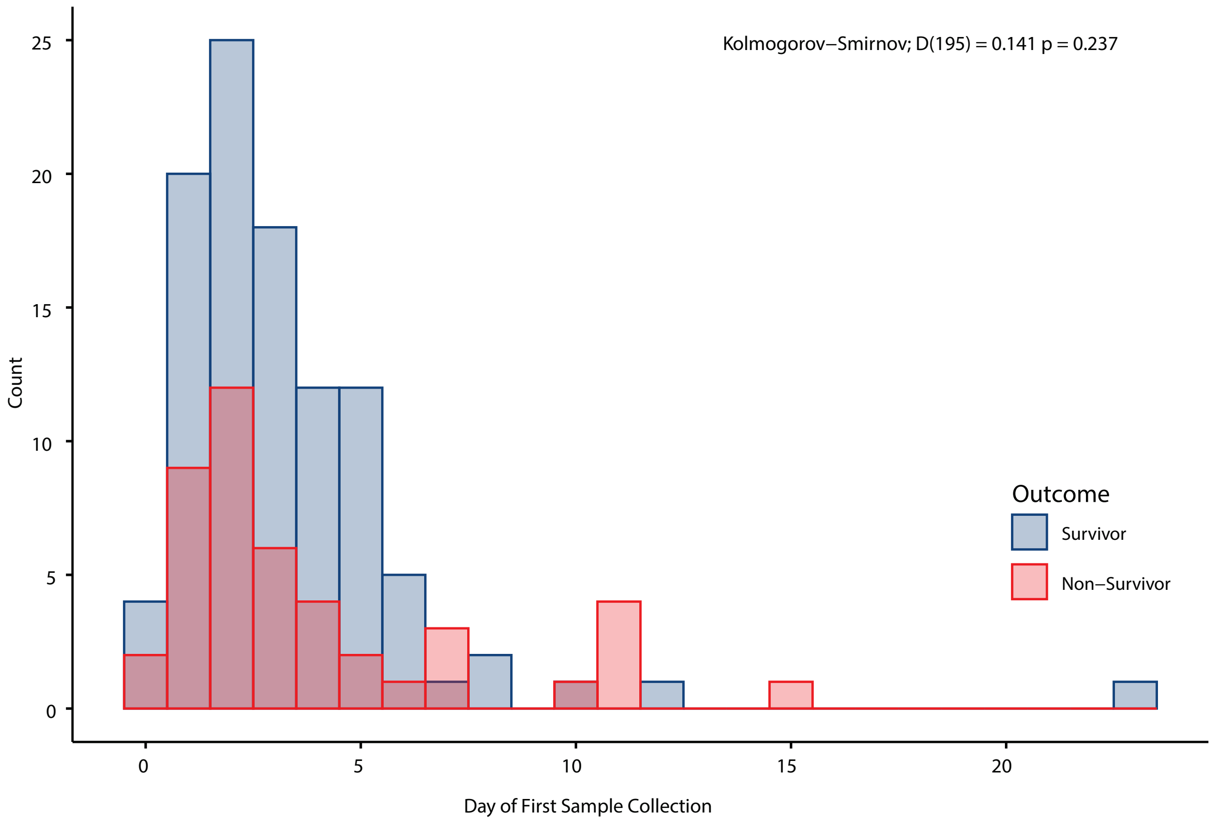

Fig. S2. Day of fecal sample collection after MICU admission.
Kolmogorov-smirnov goodness-of-fit test was used to assess the distribution of samples for both outcomes. The exact p-value was reported where the null hypothesis assumes there is no difference in the distribution of time to first sample after MICU admission.

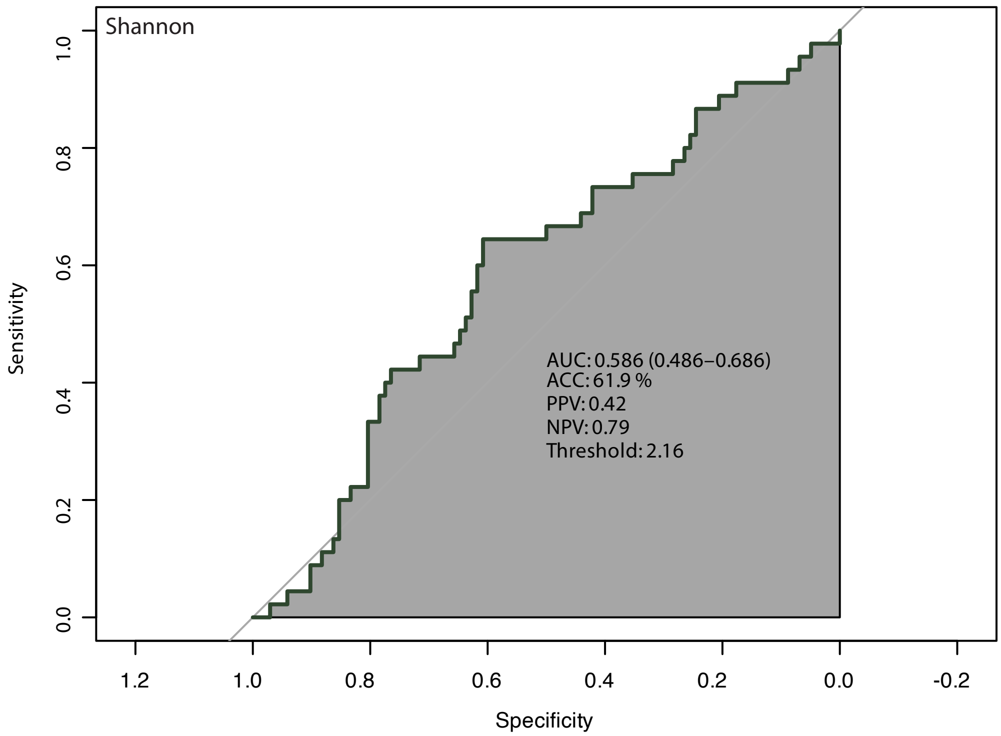

Fig. S3. Receiver operating characteristic curve for Shannon index.

The optimal threshold for alpha diversity (using the Shannon metric) was determined by the Younden Index. Using the calculated threshold Area Under the Curve (AUC), accuracy (ACC), Positive Predicted Value (PPV), Negative Predicted Value (NPV) were calculated.

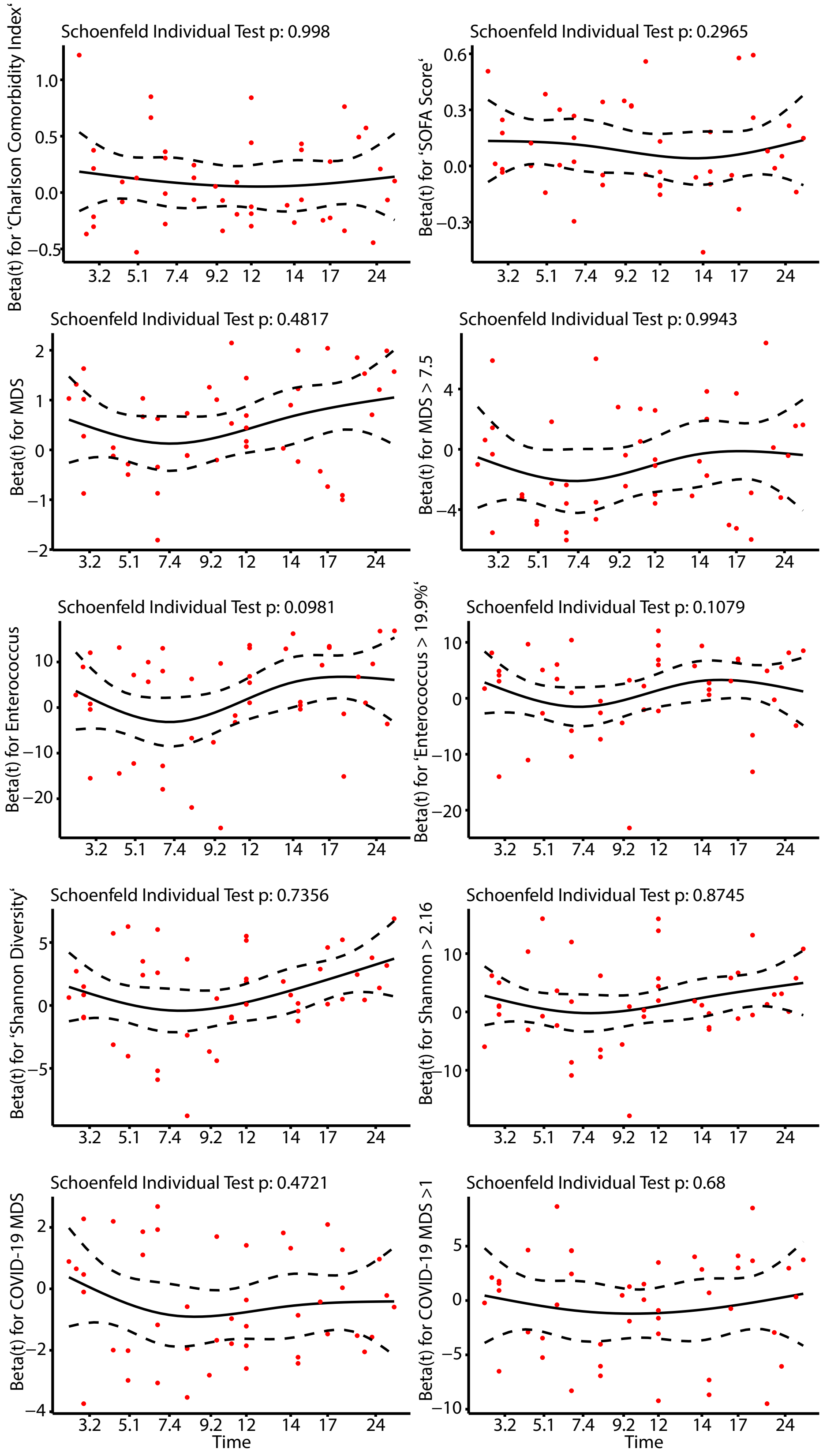

Fig. S4. Testing of Cox proportional hazard assumption with the Schoenfeld individual test for all variables within the Cox proportional hazard models.

Weighted residuals from Schoenfeld tests are plotted against time for all variables in the Coxproportional hazard model.

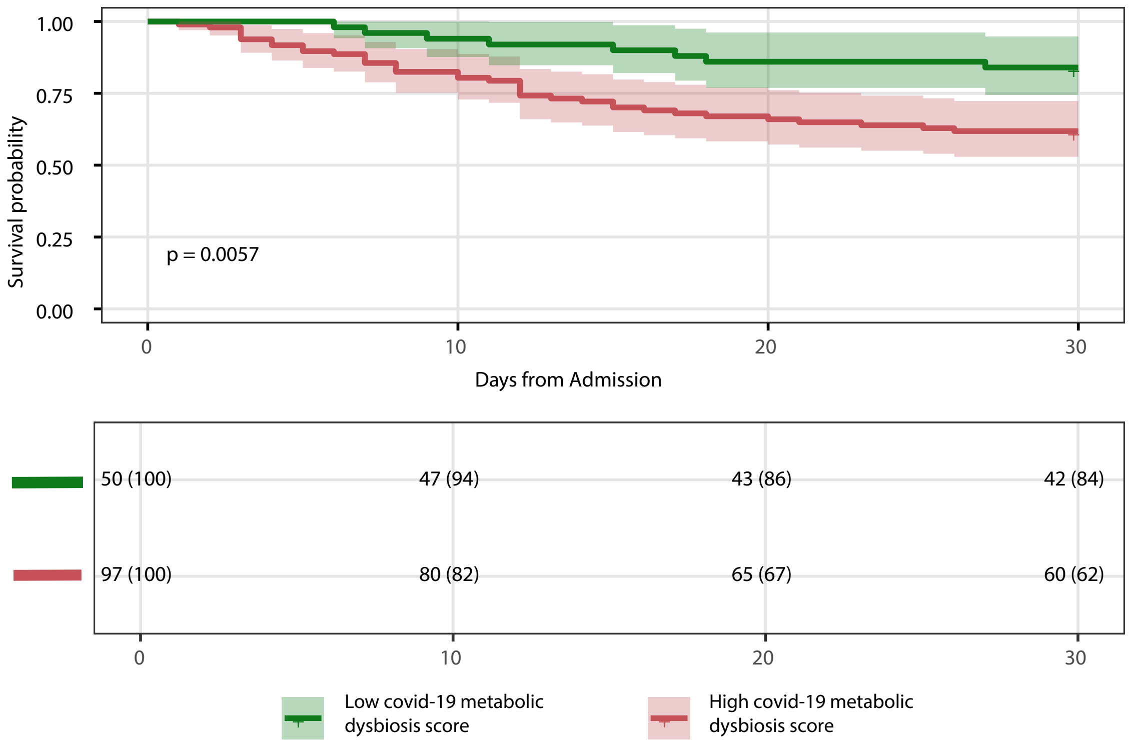

Fig. S5. Evaluating the COVID-19 metabolic dysbiosis score for predicting survival outcomes on the training cohort.

Kaplan-Meier survival analyses for high (≥2 points) and low (<2 points) COVID-19 metabolic dysbiosis scores for the training cohort. Kaplan-Meier survival analyses have time after intensive care unit admission represented on the x-axis in days with the probability of survival of each stratified group shown on the y-axis. Survival probability is shown below each curve at 10-day intervals. Groups were compared using a log-rank test to assess significance; exact p-value is reported.

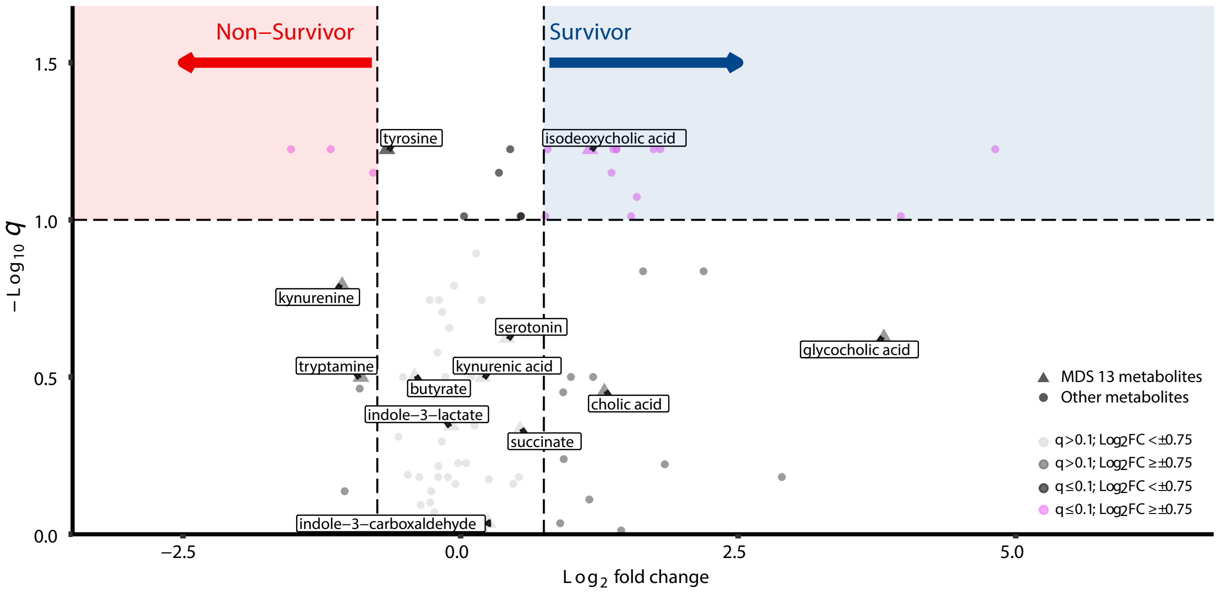

Fig. S6 MDS metabolites in relation to other relatively quantified metabolites.
Volcano analysis of qualitatively estimated metabolites, where values with a q <= 0.1are showed above the horizontal line and a Log2fold change greater than 0.75 or less than -0.75 fall in the red and blue shaded areas. Named metabolites are metabolites that make up the MDS. Wilcoxon rank-sum, two-tailed unpaired tests were performed and p-values were adjusted (q-values) for multiple comparisons via the Benjamini-Hochberg method.

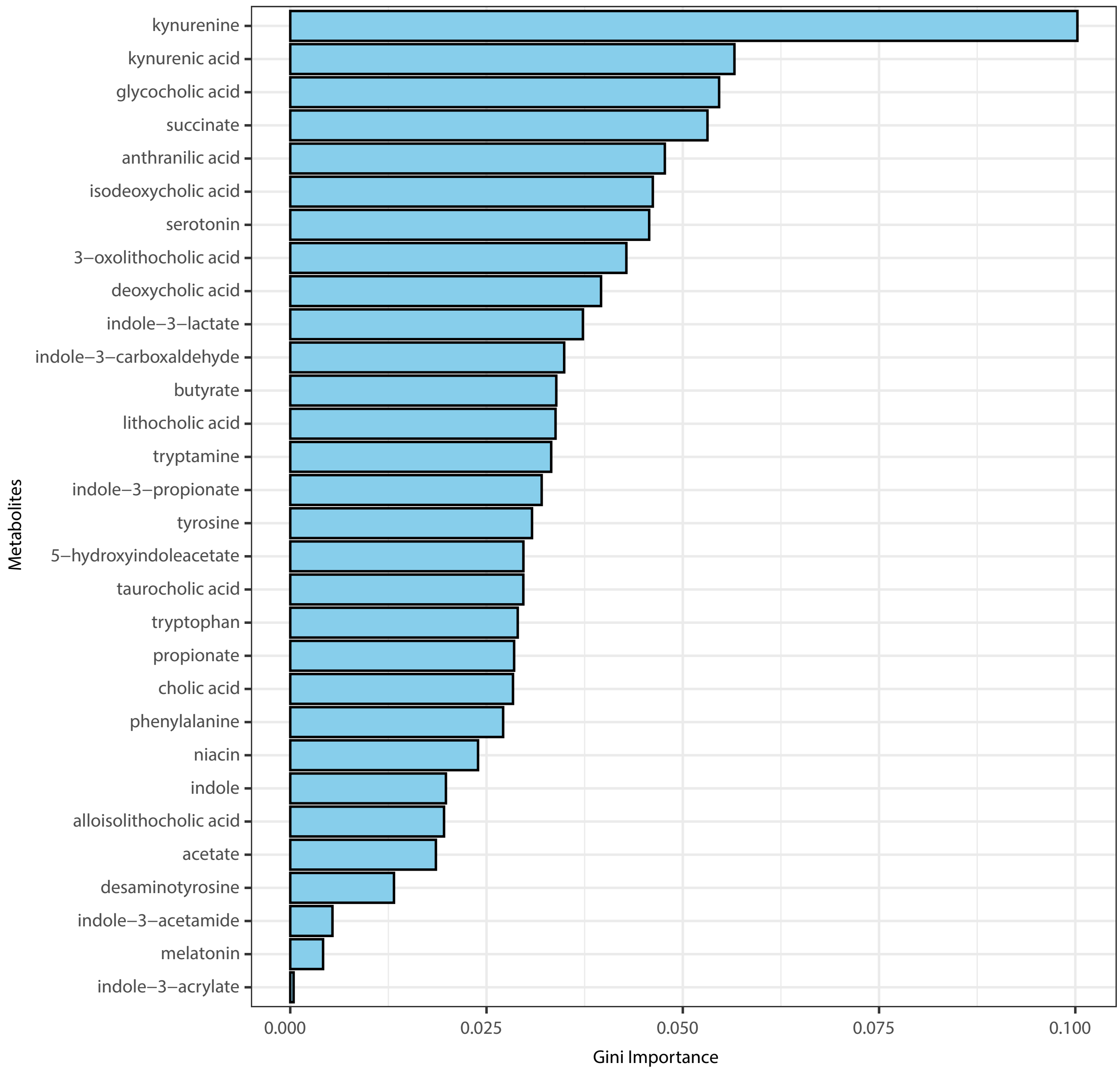

Fig. S7. Gini importance of fecal metabolites from the Random Forest model.

Gini importance from the Random Forest model is plotted in descending order from the training set.

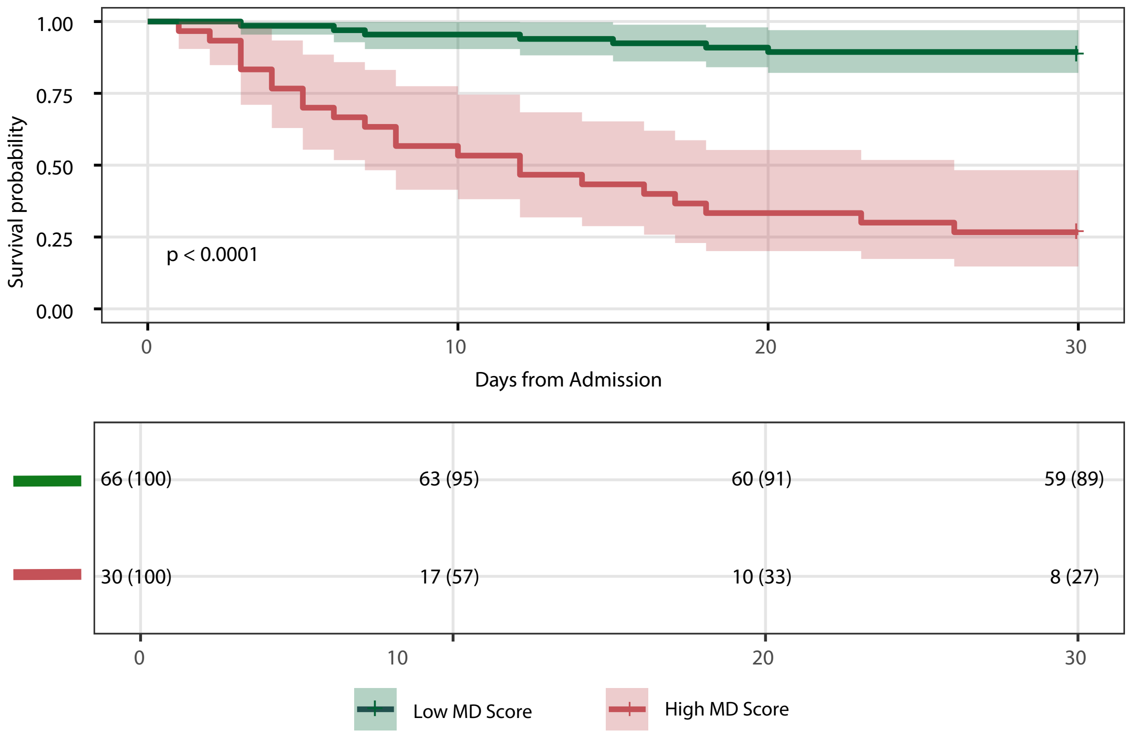

Fig. S8. Kaplan-Meier survival analyses stratifying patients with or without fecal metabolic dysbiosis according to the metabolic dysbiosis score, but only including patients with a sample collected within 3 days after MICU admission.

Kaplan-Meier survival analyses have time after intensive care unit admission represented on the x-axis in days with the probability of survival of each stratified group shown on the y-axis. Survival probability is shown below each curve at 10-day intervals. Groups were compared using a log-rank test to assess significance; exact p-value is reported.

Supplemental tables

Table S1. Description of training cohort baseline characteristics continued

	**Survivors**	**Non-survivors**	***p***
**Number of Patients**	102	45	
**Primary admission diagnosis (%)**			0.151
Acute (on chronic) liver failure	4 (3.9)	9 (20.0)	
AMI/dysrhythmia	2 (2.0)	0 (0.0)	
CHF/cardiogenic shock	3 (2.9)	0 (0.0)	
CNS pathology	2 (2.0)	0 (0.0)	
GI hemorrhage	7 (6.9)	2 (4.4)	
Metabolic	3 (2.9)	1 (2.2)	
Other	4 (3.9)	1 (2.2)	
Post-operative observation	3 (2.9)	1 (2.2)	
Respiratory failure, AHRF	28 (27.5)	13 (28.9)	
Respiratory failure, airway compromise	10 (9.8)	1 (2.2)	
Respiratory failure, ventilatory	9 (8.8)	3 (6.7)	
Sepsis (+/- septic shock)	27 (26.5)	14 (31.1)	
**Admitted from (%)**			0.002
Cardiology	2 (2.0)	1 (2.2)	
ED	51 (50.0)	11 (24.4)	
General Medicine	16 (15.7)	8 (17.8)	
Liver	3 (2.9)	6 (13.3)	
Neurology	4 (3.9)	0 (0.0)	
Oncology	5 (4.9)	10 (22.2)	
OSH	12 (11.8)	8 (17.8)	
Surgery	8 (7.8)	1 (2.2)	
Pediatric intensive care unit	1 (1.0)	0 (0.0)	
**Secondary admission diagnosis**			
Bacterial Pneumonia (%)	28 (27.5)	12 (26.7)	1
Fungal Pneumonia (%)	2 (2.0)	1 (2.2)	1
Viral Pneumonia (%)	2 (2.0)	0 (0.0)	0.862
Chronic Obstructive Pulmonary Disease (COPD) (%)	6 (5.9)	0 (0.0)	0.227
Asthma Exacerbation (%)	2 (2.0)	0 (0.0)	0.862
Pulmonary embolism (%)	5 (4.9)	1 (2.2)	0.761
Hemoptysis (%)	1 (1.0)	0 (0.0)	1
Pancreatitis (%)	1 (1.0)	1 (2.2)	1
Infection, genitourinary system (%)	15 (14.7)	3 (6.7)	0.272
Infection, Intra-abdominal (%)	10 (9.8)	14 (31.1)	0.003
Infection, soft tissue (%)	7 (6.9)	7 (15.6)	0.177
Infection, CNS (%)	3 (2.9)	0 (0.0)	0.596
Hepatic failure acute fulminant (%)	0 (0.0)	2 (4.4)	0.17
Hepatic failure, acute on chronic (%)	6 (5.9)	6 (13.3)	0.233

Diabetic ketoacidosis (%)	2 (2.0)	3 (6.7)	0.339
Acute leukemia (%)	0 (0.0)	1 (2.2)	0.673
Cerebreal vascular accident (%)	4 (3.9)	1 (2.2)	0.976
Acute myocardial infarction (%)	2 (2.0)	0 (0.0)	0.862
Diffuse alveolar hemorrhage (%)	1 (1.0)	0 (0.0)	1
Decompensated heart failure/Pulmonary oedema (%)	17 (16.7)	2 (4.4)	0.077
Pleural effusion (%)	1 (1.0)	7 (15.6)	0.001
Interstitial lung disease exacerbation (%)	1 (1.0)	1 (2.2)	1
Other (%)	15 (14.7)	5 (11.1)	0.745
Angioedema (%)	1 (1.0)	0 (0.0)	1
Acute renal failure (%)	34 (33.3)	27 (60.0)	0.004
Altered mental status (%)	23 (22.5)	13 (28.9)	0.538
Hypertensive urgency (%)	1 (1.0)	0 (0.0)	1
Hypertensive emergency (%)	2 (2.0)	0 (0.0)	0.862
Endocarditis (%)	3 (2.9)	0 (0.0)	0.596
Bacteremia (%)	5 (4.9)	4 (8.9)	0.578
Gastrointestinal bleeding(%)	2 (2.0)	0 (0.0)	0.862
Hemorrhagic shock (%)	0 (0.0)	1 (2.2)	0.673
Aspiration (%)	7 (6.9)	6 (13.3)	0.338
Central line associated blood steam infection (%)	3 (2.9)	1 (2.2)	1
Prosthetic joint infection (%)	1 (1.0)	0 (0.0)	1
New onset atrial fibrillation (%)	3 (2.9)	0 (0.0)	0.596
Newly diagnosed solid malignancy (%)	1 (1.0)	1 (2.2)	1
**Charlson comorbidity index, subcategories**			
Myocardial infract (%)	6 (5.9)	3 (6.7)	1
Congestive heart failure (%)	23 (22.5)	6 (13.3)	0.285
Peripheral vascular disease (%)	1 (1.0)	3 (6.7)	0.161
Cerebrovascular disease (%)	17 (16.7)	2 (4.4)	0.077
Dementia (%)	4 (3.9)	3 (6.7)	0.764
Chronic pulmonary disease (%)	34 (33.3)	11 (24.4)	0.377
Connective tissue disease (%)	8 (7.8)	2 (4.4)	0.69
Ulcer disease (%)	2 (2.0)	0 (0.0)	0.862
Mild liver disease (%)	2 (2.0)	4 (8.9)	0.132
Diabetes (without complications) (%)	12 (11.8)	7 (15.6)	0.715
Diabetes (with end organ damage) (%)	13 (12.7)	2 (4.4)	0.216
Hemiplegia (%)	7 (6.9)	2 (4.4)	0.849
Moderate or severe renal disease (%)	14 (13.7)	3 (6.7)	0.34
Solid tumor (non-metastatic) (%)	19 (18.6)	6 (13.3)	0.583
Leukemia (%)	4 (3.9)	2 (4.4)	1
Lymphoma (%)	5 (4.9)	1 (2.2)	0.761
Moderate or severe liver disease (%)	10 (9.8)	9 (20.0)	0.152

Solid tumor (metastatic) (%)	4 (3.9)	11 (24.4)	<0.001
AIDS (%)	2 (2.0)	0 (0.0)	0.862
**Diet**			
Patient on nothing by mouth (%)	27 (26.5)	20 (44.4)	0.05

Categorical variables were compared using two-tailed, chi-squared test, while continuous variables were compared using the unpaired, two sample t-test, two-tailed. Unadjusted p-values are presented as exact values.

AIDS: acquired immune deficiency syndrome, AHRF: acute hypoxic respiratory failure, AMI: acute myocardial infarction, CHF: congestive heart failure, CNS: central nervous system, ED: emergency department, GI: gastro-intestinal, OSH: outside hospital.

Table S2. Cox proportional hazard ratio model including Shannon Diversity.

Characteristic	Hazard Ratio	95% confidence interval	p-value
**Continuous**			
Charlson Comorbidity Index	1.08	0.97 - 1.19	0.166
SOFA Score	1.14	1.05 - 1.23	0.001
Shannon diversity	0.95	0.72 – 1.25	0.719
**Dichotomous**			
Charlson Comorbidity Index	1.07	0.97 - 1.18	0.200
SOFA Score	1.12	1.03 - 1.21	0.005
Shannon diversity	0.95	0.72 – 1.25	0.719
High diversity (Shannon >2.16)			
Low diversity (Shannon≤ 2.16)	1.61	0.83 – 3.14	0.158

Unadjusted p-values were obtained from a likelihood ratio test and reported as exact values.
SOFA: Sequential Organ Failure Assessment.

Table S3. Cox proportional hazard ratio model including Enterococcus relative abundance

Characteristic	Hazard Ratio	95% confidence interval	p-value
**Continuous**			
Charlson Comorbidity Index	1.07	0.96 – 1.18	0.221
SOFA Score	1.14	1.06 – 1.23	5.83e-04
Enterococcus relative abundance	1.73	0.8 – 3.7	0.161
**Dichotomous**			
Charlson Comorbidity Index	1.06	0.96 – 1.18	0.233
SOFA Score	1.14	1.06 – 1.23	5.39e-04
Enterococcus high/low			
No domination (relative abundance <19.9%)	-	-	
Domination (relative abundance>19.9%)	1.61	0.88 – 2.94	0.121

Unadjusted p-values were obtained from a likelihood ratio test and reported as exact values.
SOFA: Sequential Organ Failure Assessment

Table S4. Log2 fold change in fecal bile acid, SCFA and tryptophan metabolite concentrations between survivors and non-survivors.

Metabolite	Log2 fold change	P adjusted
12-oxochenodeoxycholic acid	1.64	0.15
12-oxolithocholic acid	1.74	0.06
2-methylbutyrate	-0.10	0.22
3-aminoisobutyrate	-0.02	0.59
3-aminoisobutyrate2	0.14	0.33
3-deoxycholic acid	1.16	0.77
3-oxocholic acid	0.92	0.35
3-oxodeoxycholic acid or 3-oxochenodeoxycholic acid	1.38	0.06
3-oxolithocholic acid	0.54	0.10
5-aminovalerate	0.21	0.97
5-hydroxyindoleacetate	1.45	0.97
7-oxodeoxycholic acid	1.54	0.10
7-oxolithocholic acid or 6-oxolithocholic acid	0.45	0.06
acetate	-0.14	0.41
alanine	-0.24	0.85
allocholic acid	0.09	0.32
alloisolithocholic acid	-0.52	0.32
allolithocholic acid	3.97	0.10
alpha-muricholic acid	0.35	0.07
anthranilic acid	-1.04	0.73
aspartic acid	-0.11	0.66
benzoic acid	-0.17	0.50
beta-muricholic acid	0.78	0.06
biotin	1.19	0.32
butyrate	-0.41	0.32
chenodeoxycholic acid	1.59	0.09
cholic acid	1.30	0.35
cysteine	-0.20	0.60
deoxycholic acid	1.36	0.07
desaminotyrosine	-0.50	0.97
dopamine	-0.14	0.32
gamma-muricholic acid	0.14	0.13
glutamic acid	-0.37	0.90
glycine	-0.56	0.49
glycochenodeoxycholic acid	2.19	0.15
glycocholic acid	3.81	0.24
glycodehydrocholic acid	0.37	0.22
glycodeoxycholic acid	1.80	0.06

glycohyodeoxycholic acid	0.99	0.32
glycolithocholic acid	1.40	0.06
glycoursodeoxycholic acid	4.82	0.06
hexanoate	0.13	0.45
hyodeoxycholic acid	0.03	0.10
indole	0.26	0.67
indole-3-carboxaldehyde	0.25	0.92
indole-3-lactate	-0.08	0.45
isobutyrate	-0.17	0.20
isodeoxycholic acid	1.17	0.06
isoleucine	-0.19	0.97
isolithocholic acid	0.76	0.10
isovalerate	-0.21	0.27
kynurenic acid	0.20	0.32
kynurenine	-1.07	0.16
leucine	-0.36	0.81
lithocholic acid	1.41	0.06
lysine	-0.20	0.66
methionine	-0.26	0.73
niacin	0.49	0.23
omega-muricholic acid	0.54	0.10
p-cresol	-0.28	0.18
palmitic acid	-0.91	0.34
pantothenic acid	0.73	0.29
phenol	0.90	0.92
phenylalanine	-0.27	0.79
proline	-0.05	0.69
propionate	-0.06	0.16
serotonin	0.42	0.24
succinate	0.54	0.46
tauro-alpha-muricholic acid or tauro-beta-muricholic acid	0.53	0.66
taurochenodeoxycholic acid	0.93	0.57
taurocholic acid	1.84	0.60
taurodeoxycholic acid	-0.19	0.18
taurohyodeoxycholic acid	0.47	0.69
taurolithocholic acid	-1.53	0.06
tauroursodeoxycholic acid	2.89	0.66
tryptamine	-0.90	0.32
tryptophan	-1.17	0.06
tryptophol	-0.37	0.66
tyramine	0.05	0.59

tyrosine	-0.66	0.06
ursodeoxycholic acid	-0.79	0.07
valerate	0.19	0.18
valine	-0.48	0.65

Log2 fold change values were determined by dividing the mean value of the survivor group by the non-survivor group and then taking the log2 of that value. Wilcoxon rank-sum, two-tailed unpaired tests were performed and p-values were adjusted (q-values) for multiple comparisons via the Benjamini-Hochberg method.

Table S5. Cox proportional hazard ratio model including the COVID-19 metabolic dysbiosis score.

**Characteristic**	**Hazard Ratio**	**95% confidence interval**	**p-value**
**Continuous**			
Charlson Comorbidity Index	1.07	0.97 - 1.19	0.159
SOFA Score	1.12	1.04 - 1.21	0.004
COVID-19 metabolic dysbiosis score	1.14	0.91 - 1.43	0.257
**Dichotomous**			
Charlson Comorbidity Index	1.08	0.97 – 1.19	0.150
SOFA Score	1.12	1.04 – 1.21	0.003
COVID-19 metabolic dysbiosis score			
Low score (< 2)	-	-	
High score (≥ 2)	2.08	0.94 – 4.59	0.070

Unadjusted p-values were obtained from a likelihood ratio test and reported as exact values.
SOFA: Sequential Organ Failure Assessment.

Table S6. Best performing parameters for multivariate models after tuning

Model	Best Parameters
Logistic regression	'C': 0.01, 'l1_ratio': 0.25, 'penalty': 'elasticnet', 'solver': 'saga'
Random forest	'bootstrap': True, 'class_weight': 'balanced', 'max_depth': None, 'max_features': 0.3, 'min_samples_leaf': 4, 'min_samples_split': 15, 'n_estimators': 1000
Extreme gradient boosting	'colsample_bytree': 0.8, 'learning_rate': 0.01, 'max_depth': 2, 'min_child_weight': 3, 'n_estimators': 500, 'subsample': 0.8

Table S7. Threshold concentrations, beta-coefficients and direction associated with non-survival of fecal metabolite used to construct the MDS

Compound	Beta-coefficient	Direction	Optimal cutpoint (mM)
kynurenine	0.71	>=	0.00101
glycocholic acid	0.65	<=	0.00212
succinate	0.61	>=	0.32000
tryptamine	0.54	>=	0.00422
kynurenic acid	0.42	<=	0.00317
tyrosine	0.41	>=	0.02702
cholic acid	0.38	<=	0.01939
serotonin	0.38	<=	6.5e-05
indole-3-propionate	0.35	<=	0.00016
isodeoxycholic acid	0.34	<=	0.00386
indole-3-lactate	0.33	>=	0.00088
butyrate	0.33	<=	0.42500
indole-3-carboxaldehyde	0.29	<=	0.00167
3-oxolithocholic acid	0.21	<=	0.02101
5-hydroxyindoleacetate	0.20	<=	0.00020
alloisolithocholic acid	-0.18	<=	0.00276
deoxycholic acid	0.16	<=	0.00419
taurocholic acid	0.15	>=	0.00128
propionate	0.14	<=	2.04500
melatonin	0.13	<=	6e-05
lithocholic acid	0.09	<=	0.05877
anthranilic acid	0.08	<=	2.5e-05
tryptophan	0.08	>=	0.25861
acetate	-0.08	<=	9.37500
phenylalanine	0.06	>=	0.99189
niacin	0.01	<=	0.00659
indole-3-acrylate	0.01	<=	0.02472
indole	-0.01	<=	0.00104
desaminotyrosine	0.00	<=	0.05273

Optimal thresholds of fecal metabolite concentration (mM) were established by optimizing the Youden index in an ROC analysis for each metabolite. Direction was established relative to the non-survivor group. To assess the variable importance of each metabolite, a ridge regression was performed to predict survival outcome.

Table S8. Description of validation cohort characteristics

	Survivors	Non-survivors	*p*
Number of Patients	34	15	
**Baseline characteristics**			
Age (median [IQR])	63.50 [53.50, 71.00]	62.00 [57.50, 72.00]	0.641
Male (%)	14 (41.2)	8 (53.3)	0.633
Race (%)			0.167
African American	24 (70.6)	6 (40.0)	
More than one race	1 (2.9)	1 (6.7)	
Other	0 (0.0)	1 (6.7)	
White, Hispanic	1 (2.9)	0 (0.0)	
White, non-Hispanic	8 (23.5)	7 (46.7)	
**Clinical Characteristics**			
Charlson Comorbidity Index (median [IQR])	5.00 [3.00, 6.75]	6.00 [5.00, 7.50]	0.092
Body Mass Index (median [IQR])	28.31 [23.63, 36.07]	32.28 [25.56, 37.39]	0.529
Acute respiratory distress syndrome (%)	4 (11.8)	5 (33.3)	0.162
Sepsis (%)	25 (73.5)	14 (93.3)	0.23
SOFA Score (median [IQR])	7.50 [4.25, 10.00]	11.00 [8.00, 14.00]	0.012
APACHE II Score (median [IQR])	22.50 [16.25, 28.75]	29.00 [24.00, 30.00]	0.049
**Antibiotics**			
Penicillins (%)	5 (14.7)	0 (0.0)	0.291
Cephalosporins (%)	19 (55.9)	10 (66.7)	0.695
Carbapenems (%)	1 (2.9)	1 (6.7)	1
Vancomycin (%)	15 (44.1)	8 (53.3)	0.775
Metronidazole (%)	8 (23.5)	3 (20.0)	1
Macrolides (%)	8 (23.5)	2 (13.3)	0.666
Quinolones (%)	1 (2.9)	0 (0.0)	1
Other Antiobiotics (%)	2 (5.9)	1 (6.7)	1
Clindamycin (%)	0 (0.0)	1 (6.7)	0.671
Aminoglycosides (%)	2 (5.9)	2 (13.3)	0.755
Doxycycline (%)	2 (5.9)	2 (13.3)	0.755
Trimethoprim-Sulfamethoxazole (%)	5 (14.7)	1 (6.7)	0.75
Rifaximin (%)	2 (5.9)	3 (20.0)	0.321
**Primary admission diagnosis (%)**			0.2
Acute (on chronic) liver failure	3 (8.8)	0 (0.0)	
CNS pathology	0 (0.0)	1 (6.7)	
GI hemorrhage	2 (5.9)	0 (0.0)	
Post-operative observation	1 (2.9)	0 (0.0)	
Respiratory failure, AHRF	11 (32.4)	6 (40.0)	
Respiratory failure, airway compromise	3 (8.8)	0 (0.0)	
Respiratory failure, ventilatory	4 (11.8)	0 (0.0)	

Sepsis (+/- septic shock)	10 (29.4)	8 (53.3)	
**Admitted from (%)**			0.81
Cardiology	2 (5.9)	1 (6.7)	
ED	13 (38.2)	4 (26.7)	
General Medicine	4 (11.8)	2 (13.3)	
Liver	1 (2.9)	1 (6.7)	
Oncology	2 (5.9)	2 (13.3)	
OSH	9 (26.5)	5 (33.3)	
Surgery	3 (8.8)	0 (0.0)	
**Secondary admission diagnosis**			
Bacterial Pneumonia (%)	9 (26.5)	5 (33.3)	0.883
Chronic Obstructive Pulmonary Disease (COPD) (%)	1 (2.9)	1 (6.7)	1
Lung/lobar collaps (%)	2 (5.9)	0 (0.0)	0.86
Pulmonary embolism (%)	1 (2.9)	0 (0.0)	1
Infection, genitourinary system (%)	8 (23.5)	4 (26.7)	1
Infection, Intra-abdominal (%)	4 (11.8)	1 (6.7)	0.975
Infection, soft tissue (%)	2 (5.9)	4 (26.7)	0.116
Infection, CNS (%)	1 (2.9)	1 (6.7)	1
Hepatic failure, acute on chronic (%)	1 (2.9)	2 (13.3)	0.452
Diabetic ketoacidosis (%)	1 (2.9)	0 (0.0)	1
Cerebreal vascular accident (%)	1 (2.9)	0 (0.0)	1
Decompensated heart failure/Pulmonary oedema (%)	6 (17.6)	3 (20.0)	1
Pleural effusion (%)	0 (0.0)	1 (6.7)	0.671
Interstitial lung disease exacerbation (%)	1 (2.9)	0 (0.0)	1
Other (%)	2 (5.9)	1 (6.7)	1
Angioedema (%)	1 (2.9)	0 (0.0)	1
Acute renal failure (%)	19 (55.9)	9 (60.0)	1
Altered mental status (%)	6 (17.6)	4 (26.7)	0.736
Hypertensive urgency (%)	1 (2.9)	0 (0.0)	1
Hypertensive emergency (%)	1 (2.9)	1 (6.7)	1
Endocarditis (%)	0 (0.0)	1 (6.7)	0.671
Bacteremia (%)	2 (5.9)	1 (6.7)	1
Aspiration (%)	2 (5.9)	3 (20.0)	0.321
Newly diagnosed solid malignancy (%)	1 (2.9)	0 (0.0)	1
**Charlson comorbidity index, subcategories**			
Myocardial infract (%)	0 (0.0)	1 (6.7)	0.671
Congestive heart failure (%)	8 (23.5)	5 (33.3)	0.715
Peripheral vascular disease (%)	2 (5.9)	0 (0.0)	0.86
Cerebrovascular disease (%)	2 (5.9)	3 (20.0)	0.321
Dementia (%)	34 (100.0)	15 (100.0)	NA

Chronic pulmonary disease (%)	13 (38.2)	4 (26.7)	0.647
Connective tissue disease (%)	5 (14.7)	1 (6.7)	0.75
Ulcer disease (%)	1 (2.9)	0 (0.0)	1
Mild liver disease (%)	1 (2.9)	1 (6.7)	1
Diabetes (without complications) (%)	8 (23.5)	0 (0.0)	0.102
Diabetes (with end organ damage) (%)	3 (8.8)	4 (26.7)	0.229
Hemiplegia (%)	2 (5.9)	0 (0.0)	0.86
Moderate or severe renal disease (%)	6 (17.6)	3 (20.0)	1
Solid tumor (non-metastatic) (%)	4 (11.8)	2 (13.3)	1
Leukemia (%)	1 (2.9)	1 (6.7)	1
Lymphoma (%)	0 (0.0)	1 (6.7)	0.671
Moderate or severe liver disease (%)	4 (11.8)	3 (20.0)	0.752
Solid tumor (metastatic) (%)	1 (2.9)	3 (20.0)	0.149
AIDS (%)	2 (5.9)	0 (0.0)	0.86
**Diet**			
**Patient on nothing by mouth (%)**	10 (29.4)	5 (33.3)	1

Categorical variables were compared using two-tailed, chi-squared test, while continuous variables were compared using the unpaired, two sample t-test, two-tailed. Unadjusted p-values are presented as exact values.

AIDS: acquired immune deficiency syndrome, AHRF: acute hypoxic respiratory failure, AMI: acute myocardial infarction, APACHE: Acute Physiology and Chronic Health Evaluation, CHF: congestive heart failure, CNS: central nervous system, ED: emergency department, GI: gastro-intestinal, IQR: inter quartile range, OSH: outside hospital, SOFA: Sequential Organ Failure Assessment.

Table S9. Cox proportional hazard ratio model including the Metabolic Dysbiosis Score in the training cohort only including patients with a sample collected within 3 days after admission to the intensive care unit.

Characteristic	Hazard Ratio	95% confidence interval	p-value
**Continuous**			
Charlson Comorbidity Index	1.11	0.98 – 1.26	0.1
SOFA Score	1.11	1.01 – 1.22	0.036
Metabolic Dysbiosis Score	1.79	1.46 – 2.18	1.19e-08
**Dichotomous**			
Charlson Comorbidity Index	1.08	0.96 – 1.22	0.183
SOFA Score	1.05	0.95 – 1.15	0.347
Metabolic Dysbiosis Score			
Low Metabolic dysbiosis score (<7.5)	-	-	
High Metabolic dysbiosis score (>7.5)	10.8	4.45 – 26.2	1.41e-07

Unadjusted p-values were obtained from a likelihood ratio test and reported as exact values. SOFA: Sequential Organ Failure Assessment.
